# Subunits of the PBAP Chromatin Remodeler Are Capable of Mediating Enhancer-Driven Transcription in *Drosophila*

**DOI:** 10.3390/ijms22062856

**Published:** 2021-03-11

**Authors:** Yulii V. Shidlovskii, Oleg V. Bylino, Alexander V. Shaposhnikov, Zaur M. Kachaev, Lyubov A. Lebedeva, Valeria V. Kolesnik, Diego Amendola, Giovanna De Simone, Nadia Formicola, Paul Schedl, Filomena Anna Digilio, Ennio Giordano

**Affiliations:** 1 Department of Gene Expression Regulation in Development, Institute of Gene Biology, Russian Academy of Sciences, 119334 Moscow, Russia; bylino@gmail.com (O.V.B.); shaldr23@gmail.com (A.V.S.); k-z-m@mail.ru (Z.M.K.); ll78@yandex.ru (L.A.L.); valeriakolesnik18@yandex.ru (V.V.K.); pschedl@princeton.edu (P.S.); 2 Center for Genetics and Life Science, Sirius University of Science and Technology, 354340 Sochi, Russia; 3 Department of Biology and General Genetics, Sechenov First Moscow State Medical University (Sechenov University), 119992 Moscow, Russia; 4 Department of Biology, Università di Napoli Federico II, 80138 Naples, Italy; Diego.amendola@hotmail.com (D.A.); Giodes88@gmail.com (G.D.S.); 5 Department of Sciences, Roma Tre University, 00154 Rome, Italy; 6 Institute of Research on Terrestrial Ecosystems (IRET) National Research Council (CNR), 05010 Porano, Italy; nformicola@unice.fr; 7 Institut de Biologie Valrose iBV UMR CNRS 7277, Université Côte d’Azur, 06108 Nice, France; 8 Department of Molecular Biology, Princeton University, Princeton, NJ 08544-1014, USA

**Keywords:** SWI/SNF, PBAP, chromatin remodeling, promoter, enhancer

## Abstract

The chromatin remodeler SWI/SNF is an important participant in gene activation, functioning predominantly by opening the chromatin structure on promoters and enhancers. Here, we describe its novel mode of action in which SWI/SNF factors mediate the targeted action of an enhancer. We studied the functions of two signature subunits of PBAP subfamily, BAP170 and SAYP, in *Drosophila*. These subunits were stably tethered to a transgene reporter carrying the *hsp70* core promoter. The tethered subunits mediate transcription of the reporter in a pattern that is generated by enhancers close to the insertion site in multiple loci throughout the genome. Both tethered SAYP and BAP170 recruit the whole PBAP complex to the reporter promoter. However, we found that BAP170-dependent transcription is more resistant to the depletion of other PBAP subunits, suggesting that BAP170 may play a more critical role in establishing enhancer-dependent transcription.

## 1. Introduction

The evolutionary conserved SWI/SNF class of chromatin remodelers plays essential roles in multiple processes of cell biology, from the regulation of transcription to chromosome segregation, DNA replication, and DNA repair [1,2,3]. In the transcription process, SWI/SNF is important for a local increase in DNA template accessibility by physical remodeling of chromatin structure [4,5,6]. ATPase activity of the central subunit of the complex mediates nucleosome sliding, eviction or disassembly [7,8,9].

In *Drosophila*, as in multiple species, the SWI/SNF remodeler, the Brahma complex, exists in two different forms: BAP (BAF in human and SWI/SNF in yeast) and PBAP (PBAF in human and RSC in yeast) [10]. A common core complex includes Brahma (BRM, SMARCA2/4 in human and STH1/SNF2 in yeast), Moira (MOR, SMARCC1/2 in human and SWI3/RSC8 in yeast), and Snr1 (SMARCB1 in human and SNF5/SFH1 in yeast) and can associate with the distinctive subunit OSA (ARID1A/B in human and SWI1 in yeast) to form the BAP complex or, alternatively, with Polybromo (PB, PBRM1 in human and RSC1/2/4 in yeast), BAP170 (ARID2 in human and RSC9 in yeast), and SAYP (PHF10 in human, no homolog in yeast) to produce the PBAP form [11,12]. The analysis of mutations affecting the signature subunits has demonstrated that BAP and PBAP execute distinct and partly antagonistic functions in transcription control and development with BAP being mainly involved in cell cycle regulation and PBAP, in signal transduction cascades and differentiation [12,13,14,15,16,17]. Interestingly, the PBAP subunits have a hierarchical role in the stability of the complex. MOR is strictly required for complex core assembly, and subunits that fail to assemble into a complex are quickly degraded [12]. BAP170 is required for the stability of PB [12,13], and the stability of BAP170, in turn, depends on SAYP [18].

The artificial tethering to DNA provides a means for studying the local functions of factors of interest. In yeast, two subunits of SWI/SNF have been tested in this way: LexA:SNF5 and LexA:SNF2 fusions acted as a potent transcription activator of the *LexAop*-carrying reporter, and activity of each fusion strongly depended on SNF2, SNF5 and SNF6 [19,20]. Similarly, a Gal4-SAYP fusion activated a UAS-carrying reporter in a PB- and BAP170-dependent way in *Drosophila* [21]. However, the reporter was located on a plasmid in these experiments.

To better characterize the local functions of the PBAP chromatin remodeler in transcription in the context of chromatin, we used a LexA:*LexAop*-mediated in vivo recruitment of the PBAP accessory subunits SAYP and BAP170. We found that the recruitment of the complex just upstream of a genome-integrated reporter core promoter is not in itself sufficient to activate transcription. Instead, transcriptional activation requires an interplay between the transgene-targeted PBAP and active genomic enhancers located nearby the reporter insertion site. This interplay results in the activation of the target promoter in a tissue-specific expression pattern characteristic of the trapped enhancer. Both SAYP and BAP170 efficiently recruit other PBAP subunits onto the promoter. However, an analysis of the enhancer capture effect mediated by targeted BAP170 or SAYP combined with in vivo depletion of specific PBAP subunits demonstrated that BAP170 is most likely responsible for mediating enhancer–promoter communication.

## 2. Results

### 2.1. In Vivo Targeting of SAYP or BAP170 to a Minimal Promoter Mediates Enhancer-Dependent Transcriptional Activation

**Figure 1:**
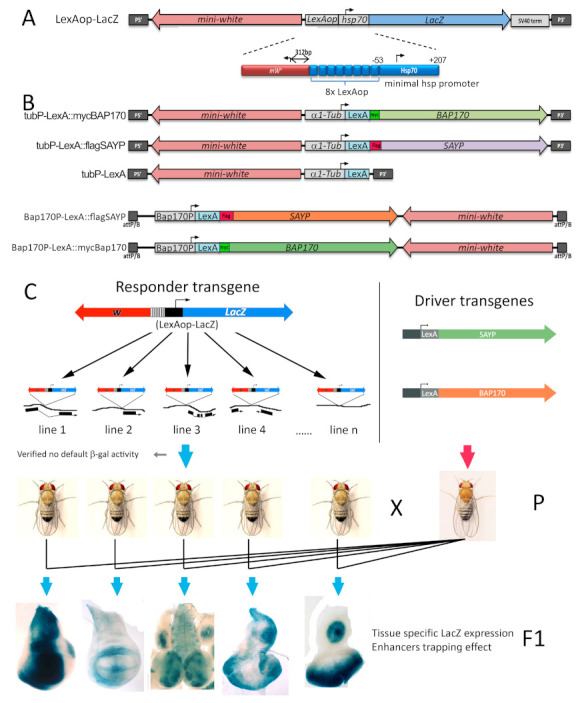
Schematic representation of the transgenes and rationale for in vivo targeting of the PBAP to a reporter promoter. (**A**) The P element-based *LexAop:LacZ* responder construct includes not only the dominant marker *white* gene, but also the *LacZ* reporter driven by the core promoter (−44 to +204) of the *Drosophila hsp70* gene. The region upstream of the *hsp70* promoter harbors 8x *Escherichia coli* LexA repressor binding elements (*LexAop* operators). (**B**) Scheme of the driver transgenes designed for LexA:BAP170, LexA:SAYP, and LexA ubiquitous expression ensured by the alpha*-*tubulin gene promoter (P element-based constructs, top) or the promoter of the *BAP170* gene (attB/P-based construct, bottom). (**C**) Rationale of the procedure (see text) used to test the effect of PBAP targeting to the core promoter of the *LexAop*-*LacZ* reporter transgenes inserted in different sites of the *Drosophila* genome.

In a whole organism, tight gene expression control in space and time is achieved via specific interactions between enhancers and core promoters. The role of the PBAP complex in such a process has never been explored in vivo, and it remains unclear how the PBAP complex functionally integrates its activity with promoter- or enhancer-bound transcription factors in the genomic context. To address this question, we decided to use an in vivo approach to reproduce the recruitment of the PBAP complex to the promoter in transgenic flies, using both SAYP and BAP170 subunits. Specific responder and driver transgenic lines were designed using the LexA/*LexAop* binary system (Figure 1). In the responder transgene *LexAop:LacZ*, a *LacZ* reporter was placed under the control of a minimal *hsp70* promoter (lacking the GAF binding sites) and 8x LexA binding sites were placed upstream of the *hsp70-LacZ* fusion (Figure 1A). This construct was used to obtain 20 independent transgenic lines, which were examined for the genomic insertion sites and orientations of the constructs. Driver transgenes for the expression of LexA:SAYP or LexA:BAP170 were prepared using either the ubiquitous alpha-tubulin promoter (P*_tub_*) or the less potent *BAP170* promoter (P*_BAP170_*) to simulate nearly physiological levels of both subunits (Figure 1B) [16]. As a control, a transgene was constructed to ensure ubiquitous expression of the LexA repressor under the P*_tub_* promoter (Figure 1B). Once transgenic stocks were established for each driver construct, we verified the proper expression of the constructs by immunostaining of larval tissues (Figure S1A,B) and checked whether the fusion proteins preserved their wild-type functions. For LexA:BAP170, we found that both types of driver transgenes were able to fully rescue mutant lethality of the *BAP170^hfl1^* null allele [16], demonstrating that the fusion is incorporated into the functional PBAP complex. The LexA:SAYP transgene did not rescue the SAYP null mutant, presumably because of the differences in expression level and its spatial pattern. However, we found that the fusion binds to the same polytene chromosome sites as the endogenous SAYP protein (Figure S2), indicating its proper recruitment to chromatin. Each *LexAop-LacZ* responder was combined with either the P*_tub_-LexA:SAYP* or P*_tub_-LexA:BAP170* transgene, and F1 third-instar larval tissues were monitored for *LacZ* expression for each cross (Figure 1C).

**Figure 2:**
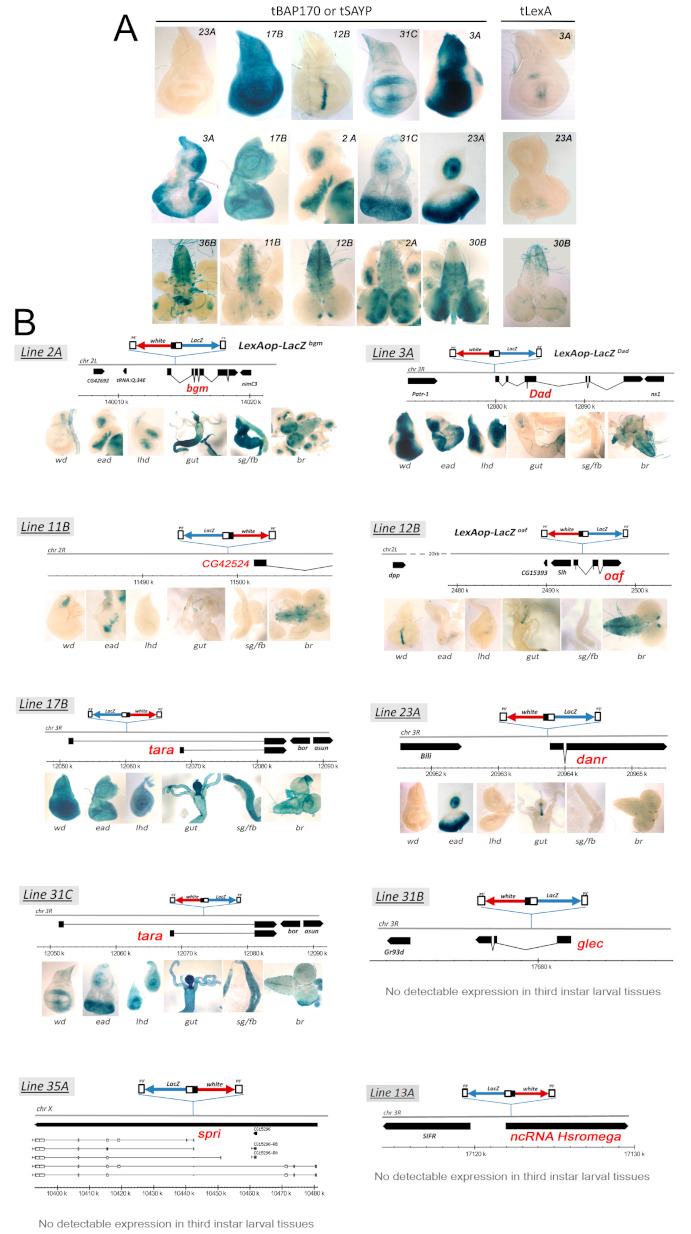
Enhancer capture by targeted BAP170/SAYP at the promoters of independent reporter transgenes. (**A**) A detailed view of the tissue-specific expression patterns obtained after introducing P*_tub_*-LexA:BAP170/SAYP to different independent lines expressing the *LexAop-LacZ* responder element in the wing discs (top), eye-antennal discs (middle), and larval brain (bottom) of L3 larvae. Note that the expression pattern was *danr*-like in 23A, *tara*-like in 17B, *dpp*-like in 12B, and *Dad*-like in line 3A. Conversely, no expression was induced by targeting the tLexA protein alone. (**B**) Genomic positions of the integrated *LexAop-LacZ* elements in the most representative lines are shown together with the corresponding P*_tub_-*BAP170-induced *LacZ* expression patterns in third-instar larval tissues. Wd, wing imaginal discs; ead, eye-antennal imaginal discs; lhd, leg-haltere discs; g, guts; sg/fb, salivary glands/fat bodies; br, larval brain.

We observed that targeted SAYP or BAP170 (tSAYP or tBAP170 hereafter) had essentially the same effect on the expression of each of the *LexAop-LacZ* responder lines we tested. Surprisingly, although the LexA fusion proteins were ubiquitously expressed under the control of the P*_tub_* promoter (Figure S1), none of the responder lines showed a corresponding ubiquitous beta-gal activity. In fact, only twelve out of the twenty *LexAop-LacZ* lines efficiently expressed the *LacZ* reporter. However, their expression pattern was not ubiquitous but instead followed a precise and reproducible tissue-specific pattern that was unique for each line (Figure 2A and Figure S3). The *LacZ* expression patterns were reminiscent of the traditional enhancer trap lines with the exception that their expression depended on tSAYP or tBAP170. Moreover, the *LacZ* expression pattern of some lines clearly reproduced the expression profile of known genes, such as *Dad* (*Daughters against dpp*, [22]), *dpp* (*decapentaplegic,* [23]), *dan* (*distal antenna*, [24]) and *tara* (*taranis*, [25]) (Figure 1C and Figure 2A).

Finally, the remaining eight *LexAop-LacZ* lines showed no appreciable *LacZ* expression in third-instar larval tissues when combined with the LexA:BAP170 or LexA:SAYP drivers. There was a possibility that an insertion occurred into silent chromatin in these eight lines; however, high levels of *mini-white* marker expression were observed in the lines without position-effect variegation, indicating that the transgenes were in euchromatin. Thus, these findings more likely indicate that only tethering tSAYP or tBAP170 to the responder transgene is not sufficient for transcriptional activation. In the control, LexA alone did not induce any expression of the responsive *LexAop-LacZ* transgenes (Figure 2A and Figure S1).

**Figure 3:**
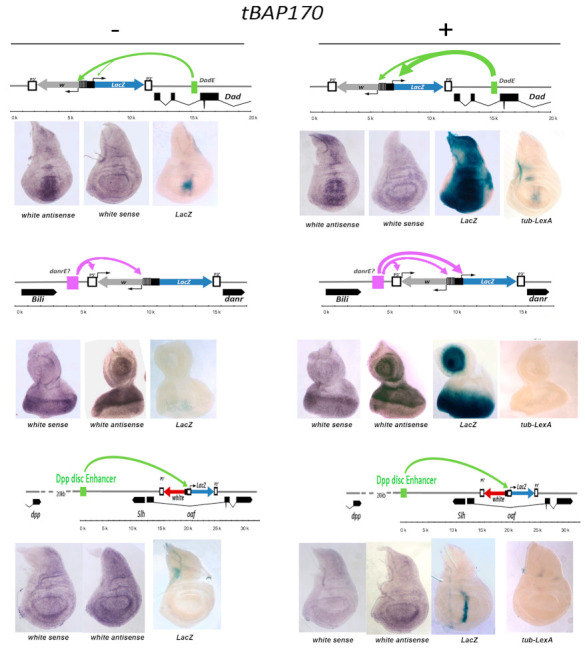
Enhancer capture by tBAP170 at the *hsp70* promoter at the *LexAop-LacZ^Dad^, LexAop-LacZ^danr^, and LexAop-LacZ^Dpp^* transgenes. Schematic drawing of the positions of the *LexAop-LacZ* responders, the enhancers, and flanking genes in the *Dad*, *danr,* and *dpp* genomic regions (top of each panel). Arrows in the genetic maps indicate the interaction between promoters and flanking enhancers; their thickness is proportional to the level of activation. Beta-gal activity is undetectable in transgenic larvae carrying the specific *LexAop-LacZ* responder alone (left of each panel), while detected in combination with P*_tub_*-*LexA:BAP170* (on the right). The negative control (P*_tub_*-*LexA*) is also given. Activity of the *hsp70* promoter was monitored by X-gal staining. The *Dad* and *danr* enhancers can spontaneously activate the *mini*-*white* and P-transposase promoters (detected by in situ hybridization with an antisense or a sense probe to the *white* gene, respectively) independently on tBAP170.

Because none of the responsive *LexAop-LacZ* transgenes showed significant beta-gal activity in the absence of tSAYP or tBAP170, we concluded that these LexA-tethered fusion proteins facilitate “enhancer capture” by the *hsp70* minimal promoter. This conclusion was supported by the analysis of the insertion sites for the responsive *LexAop-LacZ* transgenes. For example, the reporter *LexAop-LacZ^Dad^* is inserted close to the *Dad* gene (Figure 2B and Figure 3) about 5 kb upstream of the mapped *Dad* enhancer in line 3A [26]. In line 12B, the reporter *LexAop-LacZ^oaf^* is inserted within the *oaf* gene, 15 kb downstream of the *dpp* disc enhancer [27] (Figure 2B and Figure 3). As indicated in Figure 2, similar results were obtained for the other responsive *LexAop-LacZ* reporters: they are located close to genes that are normally active in larval tissues, and their expression pattern typically mimics that of the nearby gene.

To confirm that tSAYP or tBAP170 must be tethered to the *hsp70* promoter in order to facilitate “enhancer capture”, we tested whether overexpression of wild-type SAYP or BAP170 is sufficient to activate *LexAop-LacZ* expression. We found that none of the responsive *LexAop-LacZ* reporters could be activated by overexpressing wild-type BAP170 or SAYP (Figure S4). This finding demonstrates that an increase in the levels of wild types of these two PBAP subunits is not responsible for the “enhancer capture” by the *LexAop-LacZ* reporters.

In summary, these data indicate that the tSAYP/tBAP170-induced enhancer responsiveness has characteristics similar to the normal enhancer–promoter communication, (i) being achievable with both upstream and downstream enhancers and (ii) acting at a long distance.

### 2.2. Promoter-Bound PBAP Subunit Functions as a Tethering Factor for the hsp70 Minimal Promoter

The standard *hsp70* minimal promoter used in the *LexAop-LacZ* transgene (sequence from −44 to +204) has intrinsic responsiveness to many enhancers when they are located in close proximity [28,29,30], but responds less efficiently when the enhancers are at a greater distance [31]. Conversely, many other promoters used in transgenic analyses of enhancer/promoter interactions, such as the *mini-white* and P-transposase promoters, have the ability to interact at a distance as well, being naturally provided with promoter-tethering elements [29,32,33]. Accordingly, all the enhancers identified by the tBAP170/tSAYP-inducible *LexAop-LacZ* responders are fully compatible with the *hsp70* minimal promoter, but, in the absence of tBAP170/tSAYP, their interactions are presumably prevented by the lack of a tethering mechanism. This idea is supported by the pattern of expression of the *mini-white* reporter seen in the *LexAop-LacZ^Dad^* and *LexAop-LacZ^danr^* lines. In the absence of tBAP170/tSAYP, *mini-white* is expressed in a *Dad-*like pattern in the *LexAop-LacZ^Dad^* transgene insert and in a *danr-*like pattern in the *LexAop-LacZ^danr^* transgene insert (Figure 3). In contrast, the *hsp70* promoter in these two inserts is refractory to activation in the absence of tethered tBAP170 or tSAYP (Figure 3). There is also suggestive evidence of promoter competition between the P-element and *mini-white* promoters in the transgene. In the *LexAop-LacZ^danr^* transgene insert, the P-element promoter is located closer to the putative *danr* enhancer than the *mini-white* promoter. In this configuration, both sense and antisense *mini-white* transcripts are observed. In the *LexAop-LacZ^Dad^* transgene insert, the *mini-white* promoter is located closer to the *Dad* enhancer and is preferentially active. Taken together, these findings suggest that the *mini-white* and P-element promoters have intrinsic enhancer–promoter tethering elements that facilitate “enhancer capture”. In contrast, this element is missing from the *hsp70* minimal promoter.

### 2.3. Tethered BAP170/SAYP Facilitates Capture of Distant Enhancers

**Figure 4:**
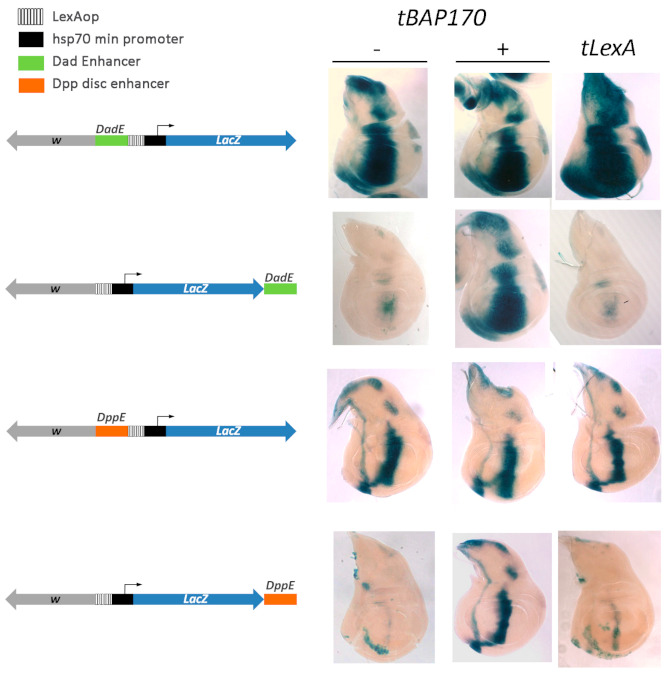
tBAP170 functions as tethering factor between the promoter and distant enhancers. Beta-Gal activity in transgenic lines with the *LexAop-hsp70-LacZ* constructs carrying the *Dad* (top) or *dpp* (bottom) enhancers. The *hsp70* core promoter can respond to both enhancers in the absence of tBAP170 when the enhancers are in close 5′ proximity (panels 1 and 3, −), but not when they are at a distance (panels 2 and 4, −). Conversely, the distant enhancers are capable of activating of the *hsp70* core promoter in the presence of tBAP170 (panel 2 and 4, +). From top to bottom, the genotypes are: *JFL-attB [DadEnh-lexAop-hsp70-LacZ]/+, JFL-attB [lexAop-hsp70-LacZ-DadEnh]/+, JFL-attB dppEnh-lexAop-hsp70-LacZ]/+, and JFL-attB [lexAop-hsp70-LacZ-dppEnh]/+.* The absence (−) or presence (+) of the P*_tub_*-*LexA:BAP170* driver is indicated. Reporter expression in the presence of tLexA is given as a control.

If tBAP170 (or tSAYP) functions as a simple tethering factor that enables the minimal *hsp70* promoter to capture distant enhancers, we would predict that relocating the enhancer closer to the *hsp70* promoter would bypass the requirement for tBAP170 (or tSAYP). Using φC31 site-specific recombination, we integrated a series of transgenes at the same attP2 site on the third chromosome. In these transgenes, the *Dad* or *dpp* enhancers were placed either just upstream of the *LexAop-hsp70-LacZ* cassettes in close proximity to the minimal *hsp70* promoter or downstream of the *LacZ* reporter about 4 kb from the promoter (Figure 4). The *Dad* and *dpp* enhancers were selected for this experiment because their positions in the genome are known [26,28].

As predicted, we found that the *Dad* and *dpp* enhancers efficiently activated *LacZ* expression when located just upstream of the minimal *hsp70* promoter (Figure 4). Moreover, *LacZ* was expressed in the appropriate enhancer pattern even in the absence of the tBAP170. Thus, the minimal promoter does not require the tBAP170 protein to interact with these two enhancers when they are located in close proximity. A completely different result was obtained when the *Dad* and *dpp* enhancers were placed 4 kb downstream of the minimal *hsp70* promoter. In this case, the promoter could only successfully engage the *Dad* or *dpp* enhancers when tBAP170 was tethered upstream of the promoter. That tethering of the PBAP subunit, tBAP170, is required for activation of the minimal *hsp70* promoter at a distant is supported by the finding that empty tLexA does not stimulate enhancer-dependent LacZ expression (Figure 4). Similar results were obtained for tSAYP. Therefore, tBAP170 and tSAYP mediate interaction between distant enhancers and the *hsp70* minimal promoter.

### 2.4. Tethered PBAP Subunit Recruits the Whole Complex onto the hsp70 Promoter

To gain insight into the molecular mechanism of action of the tethered subunits, the transgenic locus was analyzed in ChIP experiments. We used the flies that carried the *LexAop-LacZ^Dad^* responder and P*_tub_*-*LexA:BAP170* driver. Flies carrying P*_tub_*-*LexA:SAYP* had a strongly reduced viability, and we consequently could not collect enough material for ChIP experiments. Because the *Dad* gene is expressed in several larval tissues [34], whole L3 larvae were used for the ChIP experiments.

**Figure 5:**
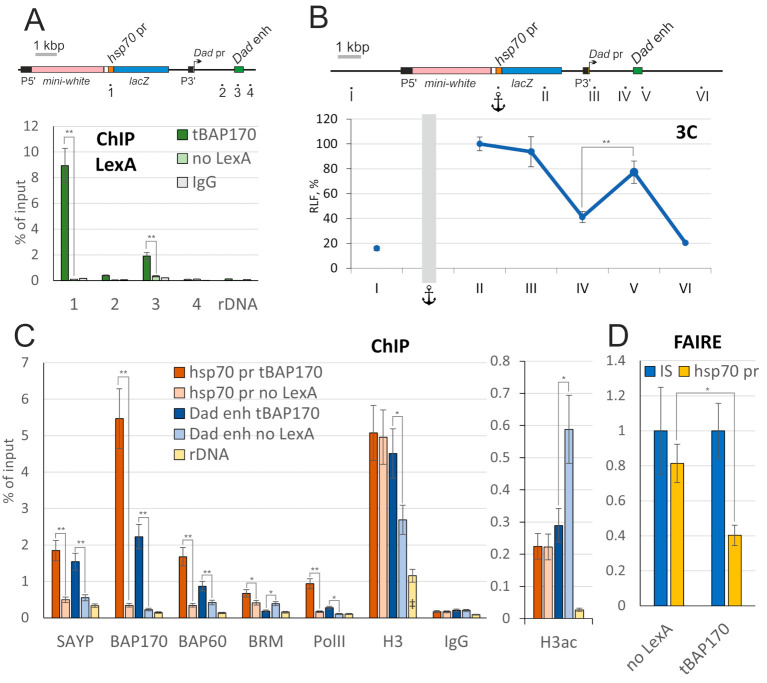
Analysis of the chromatin state in the *lexAop-LacZ^Dad^* responder line expressing the LexA:BAP170 fusion. (**A**) ChIP profile of LexA:BAP170 fusion binding along the transgenic *Dad* locus (a scheme is given at the top). Loci 1–4 indicate the positions in the transgenic *Dad* locus. IgG (pre-immune immunoglobulin) and rDNA (ribosomal 28S RNA gene) were used as negative controls. Flies expressing LexA:BAP170 were taken for the analysis; no-LexA flies are control flies, which did not express the LexA fusion. The results of ChIPs are presented as percentages of the input. (**B**) 3C analysis of the chromatin conformation in the transgenic *Dad* locus (a scheme is given at the top). Loci I-VI were checked for the relative ligation frequency (RLF) with the anchor located at the *hsp70* promoter. RLF is given as percent, 100% corresponding to the efficiency of anchor–point II ligation. (**C**) Recruitment of different proteins onto the *hsp70* reporter promoter, *Dad* enhancer, and rDNA. The LexA fusion and subunits of the PBAP complex are indicated at the bottom. PolII, RNA polymerase II; H3, histone H3; H3ac, acetylated H3. Flies expressing LexA:BAP170 and no-LexA flies were taken for the analysis. The level of a factor is shown as a percentage of Input; H3ac was normalized to H3. (‡) The level of H3 on rDNA is shown 10 times lower than actual. (**D**) FAIRE signal at the *hsp70* promoter relative to the control region (intergenic spacer, IS), which was set as 1. Control flies (no LexA) and flies expressing LexA:BAP170 fusion were tested. Asterisks indicate significance levels: * *p* < 0.05 and ** *p* < 0.01.

The LexA:BAP170 fusion was efficiently recruited onto the transgenic *hsp70* promoter (Figure 5A). At the same time, a significant peak of LexA was additionally detected on the endogenous *Dad* enhancer. This fact raised the possibility that there may be physical contacts between the enhancer and the promoter. We used 3C analysis to test it. In the experiment shown in Figure 5B, we used a primer adjacent to the *hsp70* promoter as an anchor and measured the relative ligation frequency (RLF) to fragments along the locus. The RLF steadily dropped with the increasing genomic distance from the *hsp70* promoter. However, an increase in RLF at the *Dad* enhancer was detected (Figure 5B). Thus, the minimal *hsp70* promoter appears to interact with the *Dad* enhancer.

We also determined whether other PBAP subunits are recruited to the *hsp70* promoter by tBAP170. Endogenous SAYP and two core PBAP subunits, BAP60 and central ATPase BRM, were efficiently recruited by tBAP170 (Figure 5C). An increase in RNA polymerase II (PolII) corresponding to increased gene activity was also detected. Thus, the tethered subunits efficiently recruited the whole complex, eventually leading to PolII recruitment and gene activation. Moreover, increased levels of PBAP subunits (except for BRM) and PolII were detected on the *Dad* enhancer as well (Figure 5C), further supporting the enhancer–promoter loop formation model.

Finally, we checked whether the recruited PBAP remodeler affected the chromatin state on the *hsp70* promoter and spatially close *Dad* enhancer. Despite the presence of the PBAP core, the level of histone H3 was not decreased on the active enhancer and promoter (Figure 5C). An increase in histone H3 acetylation was similarly not detected on both elements. To check the accessibility of DNA on the *hsp70* promoter, we additionally used FAIRE analysis, which is suitable for estimating the extent of chromatin remodeling in regulatory regions [35,36]. The method showed a reliable increase in the accessibility of the endogenous heat shock-inducible promoter upon activation (Figure S5A), in accordance with previously published data [37]. However, this was not observed for the minimal *hsp70* promoter in the reporter. Instead, FAIRE analysis suggests that the transgene promoter becomes less accessible upon tBAP170 recruitment (Figure 5D), possibly because the DNA template is partly hidden by abundant tBAP170.

To estimate the effect of tSAYP on chromatin state, we used flies carrying P*_BAP170_-LexA:SAYP*, which show normal viability. No significant change upon tSAYP recruitment was observed in ChIP analysis of H3 histone and its acetylation as well as FAIRE analysis of the transgene *hsp70* promoter (Figure S5B,C). Apparently, the tethered subunits of the chromatin remodelers did not create a nucleosome-free region of accessible DNA or stimulate the imposition of an epigenetic mark of active chromatin on the *hsp70* promoter and *Dad* enhancer. This finding raises the possibility that the remodeling activity of the PBAP complex may not be needed for tBAP170/tSAYP-dependent capturing of distant enhancers.

### 2.5. tSAYP-Dependent Reporter Transcription Crucially Depends on BAP170 and the Core PBAP Subunits

Because recruitment of the several components of the PBAP complex was observed in the ChIP experiments, we tested whether PBAP subunits are important for tSAYP and tBAP170-mediated transcriptional activation. The genetic system created allowed us to use the wide collection of *Drosophila* GAL4/UAS-based tools to manipulate the genetic background and make functional tests.

**Figure 6:**
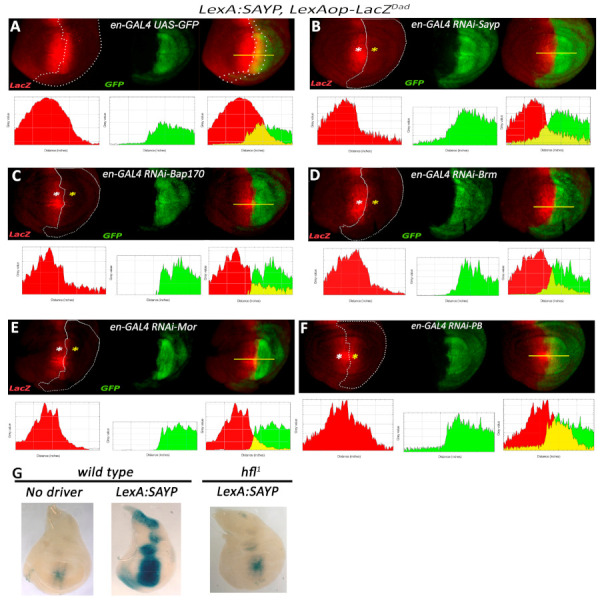
Enhancer-dependent transcriptional activation mediated by SAYP requires BAP170 and key components of the PBAP complex core. Fluorescence imaging of beta-gal (red) and GFP (green) expression in wing discs from larvae of the genotype *en-GAL4, UAS-GFP/+*; P*_BAP170_-LexA:SAYP*, *LexAop-LacZ^Dad^/+* transgenes alone (**A** control) or in combination with RNAi lines for SAYP (**B**), BAP170 (**C**), BRM (**D**), MOR (**E**), and PB (**F**). (**A**) Control wing disc from *en-Gal4*, *UAS-GFP/+*; P*_BAP170_-LexA:SAYP*, *LexAop-LacZ^Dad^/+* without RNAi. The *Dad*-like expression pattern of the SAYP-induced *LexAop-LacZ^Dad^* transgene overlaps posterior expression of en-GAL4 in a GFP-positive posterior row of cells flanking the A/P axis (cells included in the dotted line in merge image). White asterisks indicate the regions with normal *LexAop-LacZ^Dad^*transgene expression (outside the induced RNAi region), and yellow asterisks indicate the regions with expression of RNAi lines and altered target *LexAop-LacZ^Dad^* transgene activation. Genotypes: (**B**) *en-GAL4,UAS-GFP/+*; P*_BAP170_-LexA:SAYP*, *LexAop-LacZ^Dad^/UAS-RNAi-SAYP [Vdrc105946]*, (**C**) *en-GAL4,UAS-GFP/+*; P*_BAP170_-LexA:SAYP*, *LexAop-LacZ^Dad^/UAS-RNAi-BAP170 [Vdrc34582]*, (**D**) *en-GAL4,UAS-GFP/+*; P*_BAP170_-LexA:SAYP*, *LexAop-LacZ^Dad^/UAS-RNAi-Brm [Vdrc37721]*, (**E**) *en-GAL4,UAS-GFP/+*; P*_BAP170_-LexA:SAYP*, *LexAop-LacZ^Dad^/UAS-RNAi-Mor [Vdrc6969]*, (**F**) *en-GAL4,UAS-GFP/+*; P*_BAP170_-LexA:SAYP*, *LexAop-LacZ^Dad^/UAS-RNAi-PB* *[Vdrc108618]*. The plots under each wing disc panel show the intensity of staining for each color along the line at the antero/posterior boundary (yellow line in each left merged image). A dotted line encompasses the area of GFP expression. (**G**) Beta-gal activity in wing discs from larvae with the *LexAop-LacZ^Dad^* responder alone (**left**) or combined with P*_BAP170_-LexA:SAYP* in the wild type (**center**) or the *BAP170* null mutant background *hfl^1^/hfl^1^* (**right**).

To check if components of the PBAP complex are necessary for tSAYP-mediated enhancer capture, we prepared a stock that contained the P*_BAP170_-LexA:SAYP(attP2)* driver and the *LexAop-LacZ^Dad^* reporter recombined on the third chromosome together with the engrailed-GAL4 (en-GAL4) and UAS-GFP elements recombined on the second chromosome. The stock continuously expressed the *LacZ* reporter in a tSAYP-dependent *Dad*-like pattern and was used to knock down the expression of key components of the PBAP complex in the posterior compartment of the imaginal discs using the GAL4-dependent UAS-RNAi lines (Table S1). The *en*-GAL4 driver was chosen among several GAL4 drivers with *Dad* overlapping expression because it was the only one that ensured a regular growth of the third-instar imaginal discs under an RNAi regimen and was strong enough to cause an RNAi phenotype (see below). As shown in Figure 6, the overlap between the engrailed and the *Dad*-like expression patterns corresponds to a large stripe of posterior cells adjacent to the A/P boundary. In addition, all of the the RNAi lines we used had been chosen based on their ability to induce distinct morphological defects with *tubulin*-GAL4 (lethality) and *en*-GAL4 drivers (adult wings defects, our observation), as well as for their proven ability to silence their target gene expression with other GAL4 lines (see references in Table S1).

The depletion of the PBAP complex core subunit MOR or ATPase BRM was found to abolish the expression of *LacZ*, implying that their presence on the reporter promoter is necessary for enhancer-dependent transcriptional activation by tSAYP. Surprisingly, BAP170 depletion similarly abolished tSAYP-mediated reporter transcription, demonstrating that tSAYP alone is insufficient without the endogenous BAP170. This was confirmed by the finding that tSAYP failed to activate the transgene reporter in the null BAP170 *hfl^1^* mutant background (Figure 6G). Given that the depletion of BAP170 also affects the levels of PB, we additionally checked the effect of RNAi against PB (Figure 6F). We found that PB is not required for enhancer-dependent *LacZ* expression.

All of the effects described were transgene specific and were not a consequence of potential *Dad* transcription defects or functional loss of the *Dad* enhancer caused by PBAP subunit depletion. In fact, both *Dad* enhancer activity and *Dad* transcription are independent of PBAP (Figure S6), as already described for the other *Dpp* target *spalt* [17]. In conclusion, our data indicate that the PBAP subunits tested (MOR, BRM, and BAP170) are necessary for a distant enhancer to mediate the induction of expression from the transgene promoter by tSAYP.

### 2.6. tBAP170-Driven Transcriptional Activation Is Resistant to Depletion of SAYP and PBAP Core Subunits

**Figure 7:**
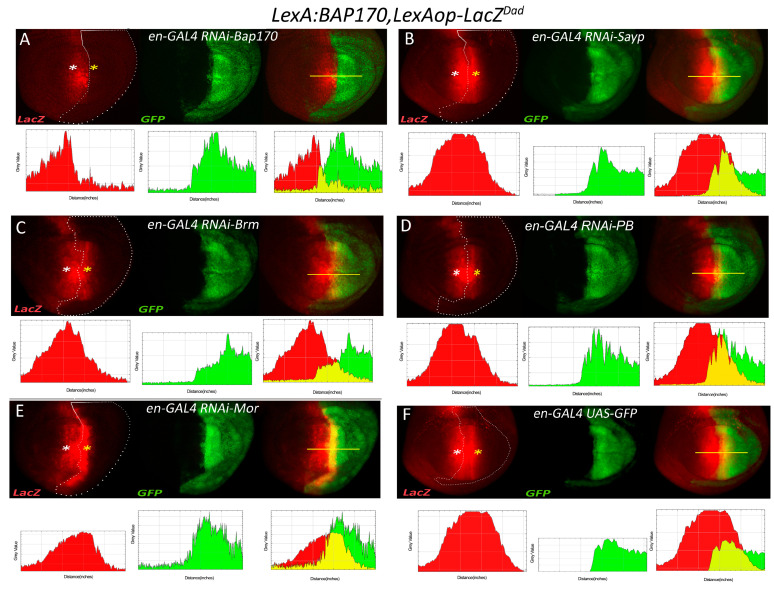
Targeted BAP170 can capture the enhancer independently by SAYP and the PBAP complex core. Fluorescence imaging of beta-gal (red) and GFP (green) expression in the wing discs from larvae of the genotype *en-GAL4, UAS-GFP/+;* P*_BAP170_-LexA:BAP170, LexAop-LacZ^Dad^/+* transgenes in combination with RNAi lines for BAP170 (**A**), SAYP (**B**), BRM (**C**), PB (**D**), and MOR (**E**). White asterisks indicate the regions with normal *LexAop-LacZ^Dad^* transgene expression (outside the region of RNAi induction), and yellow asterisks indicate the regions that overlap the RNAi line activation. Genotypes: (**A**) *en-GAL4*, *UAS-GFP/+*; P*_BAP170_-LexA:BAP170*, *LexAop-LacZ^Dad^/UAS-RNAi-BAP170 [Vdrc34582]*, (**B**) *en-GAL4*, *UAS-GFP/+*; P*_BAP170_-LexA:BAP170*, *LexAop-LacZ^Dad^/UAS-RNAi-SAYP [Vdrc105946]*, (**C**) *en-GAL4*, *UAS-GFP/+*; P*_BAP170_-LexA:BAP170*, *LexAop-LacZ^Dad^/UAS-RNAi-Brm [Vdrc37721]*, (**D**) *en-GAL4,UAS-GFP/+*; P*_BAP170_-LexA:BAP170*, *LexAop-LacZ^Dad^/UAS-RNAi-PB* *[Vdrc108618]*, (**E**) *en-GAL4*, *UAS-GFP/+*; P*_BAP170_-LexA:BAP170*, *LexAop-LacZ^Dad^/UAS-RNAi-Mor [Vdrc6969]*, (**F**) the control stock *en-GAL4*, *UAS-GFP/+*; P*_BAP170_-LexA:BAP170*, *LexAop-LacZ^Dad^/+*. The plots under each wing disc panel show the intensity of staining for each color along the line at the antero/posterior boundary (yellow line in each left merged image). A dotted line encompasses the area of GFP expression.

To determine if tBAP170-mediated enhancer capture also requires components of the PBAP complex, we prepared a stock that was similar to that described for tSAYP except that the driver expressed LexA:BAP170. The stock carried the P*_BAP170_-LexA:BAP170(attP2)* driver and the *LexAop-LacZ^Dad^* reporter recombined on the third chromosome together with the *en*-GAL4 and UAS–GFP elements recombined on the second chromosome. Both tSAYP and tBAP170 drivers were integrated in the same genomic AttP2 docking site favorable for robust expression with no position effect [23,38,39]. Unexpectedly, we found that RNAi-mediated depletion of either BRM or MOR did not cause loss of *LacZ* expression when the enhancer was captured by tBAP170 instead of tSAYP (Figure 7). Moreover, even the SAYP and PB knock down showed no effect on the expression of the transgene, which is normally active in posterior cells of the wing disc. In summary, these data indicate that tBAP170 alone may be sufficient to trigger enhancer capture independently of the core PBAP components and SAYP and that, within the PBAP complex, BAP170 may represent a subunit that functions in the tethering of enhancer–promoter interactions.

## 3. Discussion

Here, we showed that the artificial tethering of signature subunits of the PBAP chromatin remodeler onto the core *hsp70* promoter induces the expression of a downstream reporter in a way dictated by the nearby enhancer. Enhancer trapping by the promoter occurs only when a PBAP subunit is tethered to the minimal *hsp70* promoter, suggesting a crucial role in enhancer–promoter communication for the PBAP remodeler. It is known that SWI/SNF targeting to chromatin could be executed by core subunits of the complex [40,41]. However, the signature subunits seem to have greater importance for the correct recruitment of the complex [42,43]. Thus, our system appears to reproduce the endogenous mechanisms of recruitment. Indeed, multiple components of the complex were detected on the minimal *hsp70* promoter in the transgene by ChIP analysis.

The SWI/SNF remodeler is localized on promoters throughout the genome and plays an important role in the establishment of a specific nucleosome pattern on a promoter [44]. Enhancers show an even greater requirement for SWI/SNF [41,42,45,46,47,48,49,50,51,52]. SWI/SNF similarly establishes the nucleosome landscape on enhancers [48,53], and its participation in local histone acetylation has also been described [46]. Interestingly, in mammals, the PBAF subfamily shows preferable localization on promoters and BAF, on enhancers [40].

Our enhancer trapping system showed promoter specificity: the minimal *hsp70* promoter required PBAP to be activated by a distant enhancer, while the endogenous P-element and *white* promoters did not. Thus, recruited PBAP can confer an enhancer affinity on at least a certain subset of promoters. Promoter specificity of the kind is usually determined by various core promoter motifs, promoter-proximal elements, and some epigenetic signals [54,55]. The elements are recognized by a number of both DNA-binding and accessory proteins, among which architectural factors are of particular importance, mediating long-range interactions [56,57,58]. Our data indicate that PBAP is a component of this complicated system, which is necessary for establishing specific enhancer–promoter communication.

Several hypotheses could account for the role of tethered PBAP in facilitating the enhancer–promoter contacts in our system. Given the nucleosome remodeling activity of PBAP, one plausible model is that the tethered complex locally remodels nucleosomes to make the promoter more accessible to the transcriptional apparatus. However, we did not detect an increase in DNA accessibility, a decrease in nucleosome density, or an increase in histone acetylation on the promoter or even the corresponding enhancer. Previous investigations have shown that recruitment of the remodeler to a definite site is not always accompanied by local chromatin remodeling [59,60]. Moreover, the enzymatic activity of the complex is not always important for its function. In fact, Brahma was found to regulate half of its target genes in *Drosophila* through a mechanism that does not require ATPase activity [61].

In this case, a structural role in mediating enhancer–promoter interactions might be a more plausible mechanism of action. That is, the promoter-bound SWI/SNF complex interacts with enhancer-bound activators or some other factors and thus serves as a bridge in promoter–enhancer communication. Potentially supporting this idea, the SAYP subunit has previously been shown to interact with DNA-specific transcription activators [62,63].

Besides local remodeling activity and the structural role of PBAP in enhancer–promoter communication, another intriguing possibility is a direct role that ATPase activity of the complex may play in the process. There is a model of enhancer–promoter connection where the base of a chromatin loop is directly formed between the elements via protein–protein interactions. However, a loop can arise via its progressive extrusion as well [64,65]. The SWI/SNF remodeler acts as a directional DNA translocase [66] and has an intrinsic property to generate DNA loops on nucleosomes [67,68]. Loops generated in vitro are of different size, up to 1200 bp [69]. In vitro, the purified SWI/SNF complex produces loops on a polynucleosome matrix in an ATP-dependent manner, and one complex is enough to remodel a series of nucleosomes. As the complex has at least two sites to interact with different DNA sequences, it could potentially bring distantly located sites into proximity [70]. In vivo Brg1 has been found to be crucial for the formation of a loop between a regulatory element and a promoter in the *alpha-* and *beta-globin* gene loci [71,72]. At the same time, Brg1 is not involved in the formation of chromatin loops in several other loci, suggesting locus specificity for this putative mechanism. The model of SWI/SNF-mediated loop extrusion in vivo still requires further confirmation.

In this respect, it is worth noting that our knockdown experiments would seem to challenge the necessity of the whole complex for enhancer–promoter communication: BAP170 seems to drive enhancer-dependent activation independently of other subunits of the complex, while SAYP lacks this capability. However, this fact could be attributed to different affinities of these subunits for the core complex. Indeed, SAYP seems to be an optional subunit of PBAP because it is underrepresented in Brahma complex samples [18] and its gel filtration profile only partly overlaps the profiles of other Brahma subunits [21]. BAP170 as an integral component of PBAP could recruit the residual amounts of subunits after their knockdown more efficiently and could easier overcome their depletion.

The finding that BAP170 is sufficient for the enhancer capture effect to be triggered independently by core PBAP components and SAYP suggests that, within the PBAP complex, BAP170 represents the subunit that is required for conferring promoter responsiveness on the enhancer. In this scenario, LexA:SAYP can activate reporter expression only through the recruitment of BAP170, which, in turn, mediates the enhancer capture. Therefore, in LexA:SAYP experiments, a depletion of BAP170 removes the true bridging factor, while a MOR or BRM knockdown causes disassembly of the complex with final loss of BAP170 as well. Conversely, LexA:BAP170 can bypass the requirement for its natural recruiting factors due to the LexA anchor and directly makes the promoter responsive to enhancers. This model is also supported by the MOR knockdown effect, which, in fact, causes an increase in *LacZ* expression at the overlapping stripe between the *engrailed*- and *Dad*-like patterns (Figure 7C). One may assume that LexA:BAP170 exists in two states, the first assembled into PBAP complex, but defective for the activation of the *LexAop-LacZ*, and the second free form, which is not incorporated into PBAP, but is capable of activating the transgene. As MOR is a principal PBAP-stabilizing factor [12], its depletion produces more free LexA:BAP170, which can bind to the *LexAop* sites and increase reporter expression.

Interestingly, the SNF6 subunit of the remodeler in yeast can also support transcription of the reporter independently of other subunits of SWI/SNF when tethered onto the promoter via LexA [73]. However, this subunit is yeast specific, and it is unclear if BAP170 and SNF6 act the same way in transcription activation. The contribution of different SWI/SNF subunits into enhancer–promoter communication requires further investigation.

The system described here has allowed us to reveal novel aspects of the role that the remodeler plays in enhancer-dependent transcription, thus extending our knowledge about molecular mechanisms of enhancer action [74,75]. Moreover, recent data point to a localization of the remodeler on chromatin boundaries and its role in the formation of the global chromatin structure [15,76,77,78]. Our approach could be useful for studying the remodeler function on these elements to finally elucidate the full spectrum of roles that the SWI/SNF complex and its individual subunits play in gene expression regulation.

## 4. Materials and Methods

### 4.1. Plasmid Preparation

The *LexAop*-*LacZ* plasmid was prepared by cloning the BglII-*LexAop*-*hsp70*-HindIII(filled) fragment of pJFRC18-8X*LexAop*2-mCD8:GFP [39] (Addgene plasmid #26225) into the BamHI/EcoRI(filled) restriction sites of the transformation vector pCasper-AUG-beta-gal [79]. The *LexAop*-*hsp70* cassette consists of 8x (22-bp) LexA operators followed by the *hsp70* minimal core promoter from −45 to +207, thus lacking the upstream GAGA binding sites.

For the LexA:myc:BAP170 in-frame cassette, the NLS:LEXA (1–214 aa) fragment from the pBPnlsLexA::GADflUw plasmid [39] (Addgene plasmid #26232) was cloned in frame in a myc-BAP170 (2–1688 aa) cassette. For the LexA:3xFLAG:SAYP in-frame cassette, the NLS:LexA (1–214 aa) fragment from the pBPnlsLexA::GADflUw plasmid was cloned in frame in a 3xFLAG:SAYP (2–1843 aa) cassette. The P element-based transformation vectors P*_tub_*-LexA:myc:BAP170 and P*_tub_*-LexA:3xFLAG:SAYP were prepared by inserting the LexA:myc:BAP170 or LexA:3xFLAG:SAYP cassette in the pOP-118 vector, which contains the ubiquitous tubulin-1*α* gene promoter. The AttB-based BAP170 promoter-driven transgenes P*_BAP170_*-LexA:myc:BAP170 and P*_BAP170_*-LexA:3xFLAG:SAYP were prepared by inserting the LexA:myc:BAP170 and LexA:3xFLAG:SAYP cassettes in the P*_BAP170_*-AttB vector. The P*_BAP170_*-AttB vector was prepared by inserting a PCR fragment containing the BAP170 transcriptional regulatory sequences (-373/+135) [16] in the pJFRC18-8X*LexAop*2-mCD8:GFP-derived plasmid JFR-AttB. All cloning steps, maps, and plasmids are available upon request.

Full-length BAP170 cDNA clones have been previously described [16]. The full-length SAYP cDNA (LD10526) was purchased from the *Drosophila* Genome Resource Center.

Constructs for enhancer proximity tests JFL-attB [*DadEnh-lexAop-hsp70-LacZ*], JFL-attB [*lexAop-hsp70-LacZ-DadEnh*], JFL-attB [*dppEnh-lexAop-hsp70-LacZ*], and JFL-attB [*lexAop-hsp70-LacZ-dppEnh*] were prepared by cloning PCR fragments of the *Dad* and *dpp* enhancers in the JFL-attB vector. The JFL-attB vector has been prepared in our lab by modifying pJFRC18-8X*LexAop*2-mCD8::GFP as follow: (i) the NdeI/XbaI fragment corresponding to the *mini-white* cassette was recovered from pBPnlsLexA::GADflUw, filled, and cloned into the EcoRV site of pBluescript to obtain the *white*-PBS; (ii) the white cassette from pJFRC18-8X*LexAop*2-mCD8::GFP was removed by EcoRV/HindIII digestion and *mini-white* was reintroduced in the opposite orientation as a HindIII/BamHI(filled) fragment from white-PBS to obtain the JFK plasmid; (iii) the GFP cassette was then removed from JFK by BglII/XbaI digestion, and the LacZ gene was introduced as a BamHI/XbaI fragment of pCasper-AUG-*beta-gal* to obtain the final JFL-attB plasmid. Primers for cloning the Dad and dpp enhancers in JFL are described in the Supplementary Material.

### 4.2. Beta-Gal Staining, In Situ Hybridization, and Immunofluorescence

The detection of beta-gal activity for LacZ reporters was carried out according to standard protocols. Images were captured using either a Leica MZ stereomicroscope or a Reichert–Jung Polyvar microscope using incident fiber optic lights. In situ hybridizations were carried out as described in [80], with a DIG-RNA probe prepared using pBluescript-cloned PCR-amplified fragments corresponding to the *white* exon. Indirect immunofluorescences were carried out according to standard protocols. At least 20 larvae were taken for each staining. The plots in Figure 6; Figure 7 have been created using the ImageJ Analyse/plot function by measuring the pixel intensity for each color channel.

### 4.3. Transgenic Line Preparation

P element-based transgenic lines were generated by injections into *w^1118^* embryos as previously described [81], using the transposase activity provided by the helper plasmid *Turbo* Δ2–3 [82]. The chromosomal insertion sites of the *LexAop-LacZ* lines were mapped by inverse PCR with P-element end primers. The PCR fragments were then sequenced, and the insertion sites mapped via BLAST searches at Flybase. In the case of attB-based vectors, transgenic lines were prepared using φC31 attB/attP site-specific recombination by injections into embryos of the genotype *y,w, P{y[+t7.7]=nos-phiC31\int.NLS}X; P{y[+t7.7]=CaryP}attP2*. The fly stocks carrying the TM6B, Tb balancer were used in all experiments; the genotypes are indicated in the figure legends.

### 4.4. Drosophila Strains

The following GAL4 and UAS lines were used: *en-GAL4, UAS-GFP; Ms1096-GAL4; UAS-GFP; UAS-RNAi-SAYP* [Vdrc105946]; *UAS-RNAi-BAP170* [Vdrc34582]; *UAS-RNAi-Brm* [Vdrc37721]; *UAS-RNAi-Mor* [Vdrc6969]; *UAS-RNAi-PB* [Vdrc108618]. The RNAi lines were chosen on the basis of their ability to induce clear defects with *tubulin-GAL4* and *en-GAL4* drivers, as well with other GAL4 lines in the literature (see Supplementary Table S1). The *P{lacW}Dad^j1E4^* was used as a *Dad-LacZ* enhancer trap.

### 4.5. Antibodies

Primary antibodies against beta-gal (Promega, Madison, WI, USA), LexA (Millipore, Burlington, MA, USA), FLAG (Sigma, St. Louis, MO, USA), myc, GFP (Sigma), PolII CTD, histone H3 (Abcam, Cambridge, UK), H3ac (ab47915, Abcam), SAYP [83], BAP170 [18], and BAP60 [84] were used. Antibodies against fragment 652–785 of the RA form of BRM were raised in rabbits and affinity purified (Figure S7). In immunofluorescence experiments, secondary antibodies anti-mouse Alexa Fluor 568 and anti-rabbit Alexa Fluor 488 (Thermofisher, Waltham, MA, USA) were used.

### 4.6. Polytene Chromosome Analysis

Immunofluorescence analysis on polytene chromosomes was made according to the protocol [85].

### 4.7. Chromatin Immunoprecipitation, 3C and FAIRE Analysis

Whole L3 larvae were taken for analysis as described in [86]. Briefly, the larvae were collected with 20% sucrose, washed with buffer (0.7% NaCl, 0.05% Triton X-100), homogenized in a NU-1 buffer (15 mM of HEPES-KOH, pH 7.6, 10 mM of KCl, 5 mM of MgCl2, 0.1 mM of EDTA, 0.5 mM of EGTA, 0.35 M of sucrose, 1 mM of DTT, complete protease inhibitor cocktail (PIC) (Roche, Basel, Switzerland)) supplemented with 0.5% formaldehyde. The suspension was filtered through a 40-μm cell strainer, the filtrate was incubated for a total of 10 min at RT and quenched with an equimolar amount of glycine for 5 min at RT, and nuclei were centrifuged 1000g for 1 min. The pellet was washed 2 times with PBS and resuspended in 300 μL of a sonication buffer (50 mM of HEPES-KOH, pH 7.9, 140 mM of NaCl, 1 mM of EDTA, 1% Triton X-100, 0.1% deoxycholate Na, 0.1% SDS); the suspension was sonicated and centrifuged. DNA (3–10 μg) was taken for a ChIP reaction; immunoprecipitation was performed as described in [87]. For FAIRE analysis, nuclei were isolated as described above (1% formaldehyde was used) and then processed as described [88].

A 3C library was obtained essentially as described in [89] with minor modifications from [90,91,92]. Briefly, nuclei were prepared as in the ChIP experiment, the nucleoplasm was extracted, and chromatin was digested in nuclei with DpnII and then ligated using T4 DNA ligase. The crosslinks were reversed; DNA was extracted with phenol/chloroform, precipitated with ethanol, and treated with RNase. An equimolar mixture of BAC CH321-43A11 (BPRC) and the *LexAop-LacZ* plasmid was used for calibration, the data were normalized to the *RpII* locus [86]. The anchor primer hybridizes with the endogenous *hsp70* genes as well, but this hybridization was neglected because the genes are at great distance (at least several Mb) away from *Dad*. Primers for qPCR are given in the Supplementary Materials.

In these experiments, error bars indicate SDs of quadruplicate PCR technical replicates from at least three independent biological repeats.

## Data Availability

Not applicable.

## Supplementary Materials

The following are available online at https://www.mdpi.com/1422-0067/22/6/2856/s1, Figure S1: Ubiquitous expression of LexA:SAYP and LexA:BAP170. Figure S2: Colocalization of SAYP and LexA:SAYP on polytene chromosomes. Figure S3: Beta-gal activity induced in larval tissues by P*_tub_*-LexA-BAP170/SAYP in twelve out of twenty lines transgenic for the *LexAop-LacZ* reporter. Figure S4: Wild-type SAYP or BAP170 overexpression cannot induce enhancer-dependent *LexAop-LacZ* expression. Figure S5: Analysis of the chromatin state. Figure S6: *Dad* expression is independent of PBAP. Figure S7: Western blot analysis of a nuclear embryonic extract with a-BRM antibody. Supplementary Table S1: UAS-RNAi lines used in this work.
