# Supplementary material for: Subunits of the PBAP Chromatin Remodeler Are Capable of Mediating Enhancer-Driven Transcription in Drosophila
Source: Int J Mol Sci. 2021 Mar 11;22(6):2856. doi: 10.3390/ijms22062856 (PMC7999800; doi:10.3390/ijms22062856)
Supplement: Supplementary file 1 [file ijms-22-02856-s001.zip › S1_Table.docx]

|  | *Affected*  *Gene* | *RNAi Line* | Effect on *LexA:Sayp, LexAop-LacZ^Dad^*  expression | Effect on *LexA:Bap170, LexAop-LacZ^Dad^*  expression | Effect on Dad expression | Phenotype with  engrailed-GAL4  (this work) | Phenotype with  tubulin-GAL4  (this work) | Phenotypes described with other Gal4 lines |
| --- | --- | --- | --- | --- | --- | --- | --- | --- |
| PBAP COMPLEX | *mor* | *VDRC6969* | ↓ | ↑  (Discussed in the text) | N.E. | Prepupal lethal | larval/prepupal lethal | pnr-GAL4^a^  Bx-MS1096-GAL4^b^  salm-GAL4^c^  *C564‐Gal4 and Hml‐Gal4^e^*  http://flybase.org/reports/FBal0210097.html |
|  | *bap170* | *VDRC34582* | ↓ | ↓ | N.E. | Adult wing extraveins | prepupal lethal | pnr-GAL4^a^  salm-GAL4^c^  http://flybase.org/reports/FBal0198822.html |
|  | *brm* | *VDRC37721* | ↓ | N.E. | N.E. | Adult wing defects | larval/prepupal lethal | pnr-GAL4^a^  salm-GAL4^c^  insc-GAL4^d^  http://flybase.org/reports/FBal0209177.html |
|  | *e(y)3*  *(SAYP)* | *VDRC105946* | ↓ | N.E. | N.E. | Adult wing defects | larval/prepupal lethal | - |
|  | *polybromo*  *(PB)* | *VDRC108618* | N.E. | N.E. | N.E. | wt | viable | C564‐Gal4 and Hml‐Gal4^e^  bab1-Gal4^f^  http://flybase.org/reports/FBal0231490.html |

1. Mummery-Widmer, J.L.; Yamazaki, M.; Stoeger, T.; Novatchkova, M.; Bhalerao, S.; Chen, D.; Dietzl, G.; Dickson, B.J.; Knoblich, J.A. Genome-wide analysis of Notch signalling in Drosophila by transgenic RNAi. Nature 2009, 458, 987-992, doi:10.1038/nature07936.
2. Dietzl, G.; Chen, D.; Schnorrer, F.; Su, K.C.; Barinova, Y.; Fellner, M.; Gasser, B.; Kinsey, K.; Oppel, S.; Scheiblauer, S., et al. A genome-wide transgenic RNAi library for conditional gene inactivation in Drosophila. Nature 2007, 448, 151-156, doi:10.1038/nature05954.
3. Terriente-Felix, A.; de Celis, J.F. Osa, a subunit of the BAP chromatin-remodelling complex, participates in the regulation of gene expression in response to EGFR signalling in the Drosophila wing. Dev Biol 2009, 329, 350-361, doi:10.1016/j.ydbio.2009.03.010.
4. Neumuller, R.A.; Richter, C.; Fischer, A.; Novatchkova, M.; Neumuller, K.G.; Knoblich, J.A. Genome-wide analysis of self-renewal in Drosophila neural stem cells by transgenic RNAi. Cell Stem Cell 2011, 8, 580-593, doi:10.1016/j.stem.2011.02.022.
5. Bonnay, F.; Nguyen, X.H.; Cohen-Berros, E.; Troxler, L.; Batsche, E.; Camonis, J.; Takeuchi, O.; Reichhart, J.M.; Matt, N. Akirin specifies NF-kappaB selectivity of Drosophila innate immune response via chromatin remodeling. EMBO J 2014, 33, 2349-2362, doi:10.15252/embj.201488456.
6. He, J.; Xuan, T.; Xin, T.; An, H.; Wang, J.; Zhao, G.; Li, M. Evidence for chromatin-remodeling complex PBAP-controlled maintenance of the Drosophila ovarian germline stem cells. PLoS One 2014, 9, e103473, doi:10.1371/journal.pone.0103473.
