# Supplementary material for: Subunits of the PBAP Chromatin Remodeler Are Capable of Mediating Enhancer-Driven Transcription in Drosophila
Source: Int J Mol Sci. 2021 Mar 11;22(6):2856. doi: 10.3390/ijms22062856 (PMC7999800; doi:10.3390/ijms22062856)
Supplement: Supplementary file 1 [file ijms-22-02856-s001.zip › Shidlovskii Supplement ver 2.docx]

## Subunits of the PBAP chromatin remodeler are capable of mediating enhancer-driven transcription in Drosophila

## Shidlovskii YV at al.

## Supplementary Figures


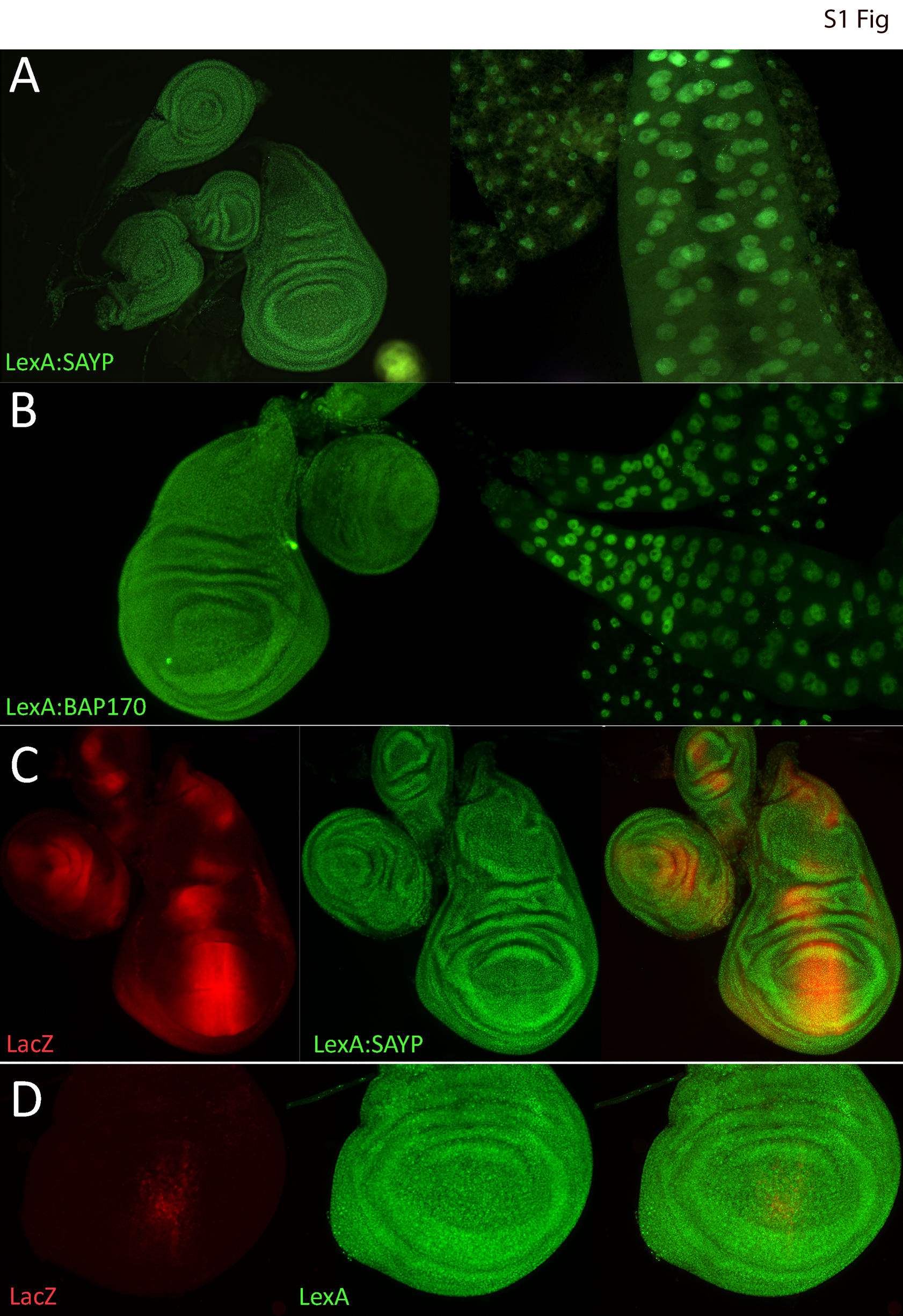


## Figure S1. Ubiquitous expression of LexA:SAYP and LexA:BAP170.

(A-B) Immunofluorescence imaging of LexA:SAYP and LexA:BAP170 show their ubiquitous expression and nuclear localization in larval tissues (wing imaginal discs are on the left and salivary glands, on the right) of the transgenic stocks carrying P*_tub_-LexA:SAYP* (A) or P*_BAP170_-LexA:BAP170* (B). a-LexA antibody was used. (C) Double immunolocalization of beta-gal (red) and LexA:SAYP (green) in imaginal discs of transgenic *LexAop-LacZ^Dad^ ,* P*_BAP170_-LexA:SAYP* larvae shows that, despite the ubiquitous LexA:SAYP expression in wing discs, the *LacZ* reporter is expressed according to the *Dad*-like pattern in a central, vertical stripe of the discs. (D) The LexA protein ubiquitously expressed from the *P_tub_-LexA* transgene cannot alone induce expression of the *LexAop-LacZ^Dad^* transgene.


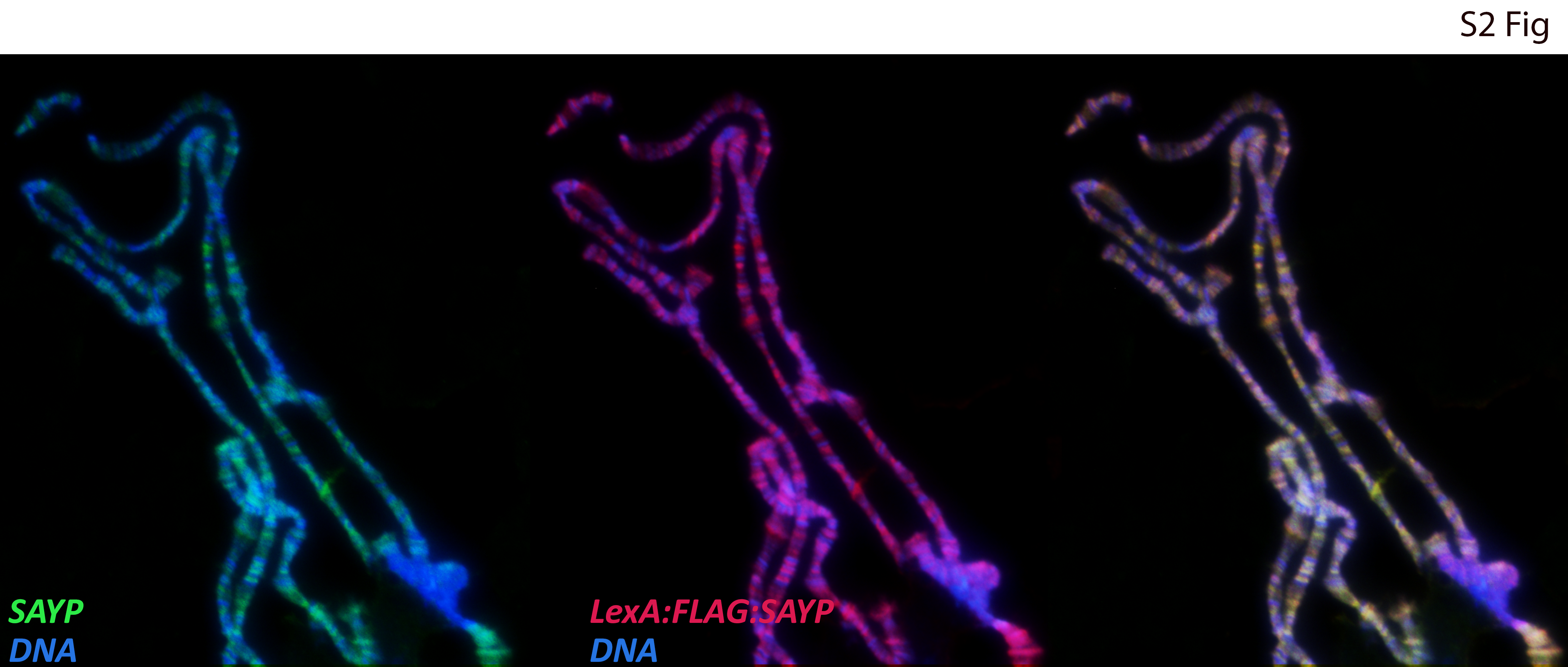


## Figure S2. Colocalization of SAYP and LexA:SAYP on polytene chromosomes.

The endogenous SAYP (anti-SAYP antibody, left panel, green) and LexA:3xFLAG:SAYP (anti-FLAG antibody, central panel, red) localize at the same discrete polytenic bands (merge on the right) in transgenic P*_BAP170_*-*LexA:FLAG:SAYP* larvae. DAPI staining (blue) marks DNA.


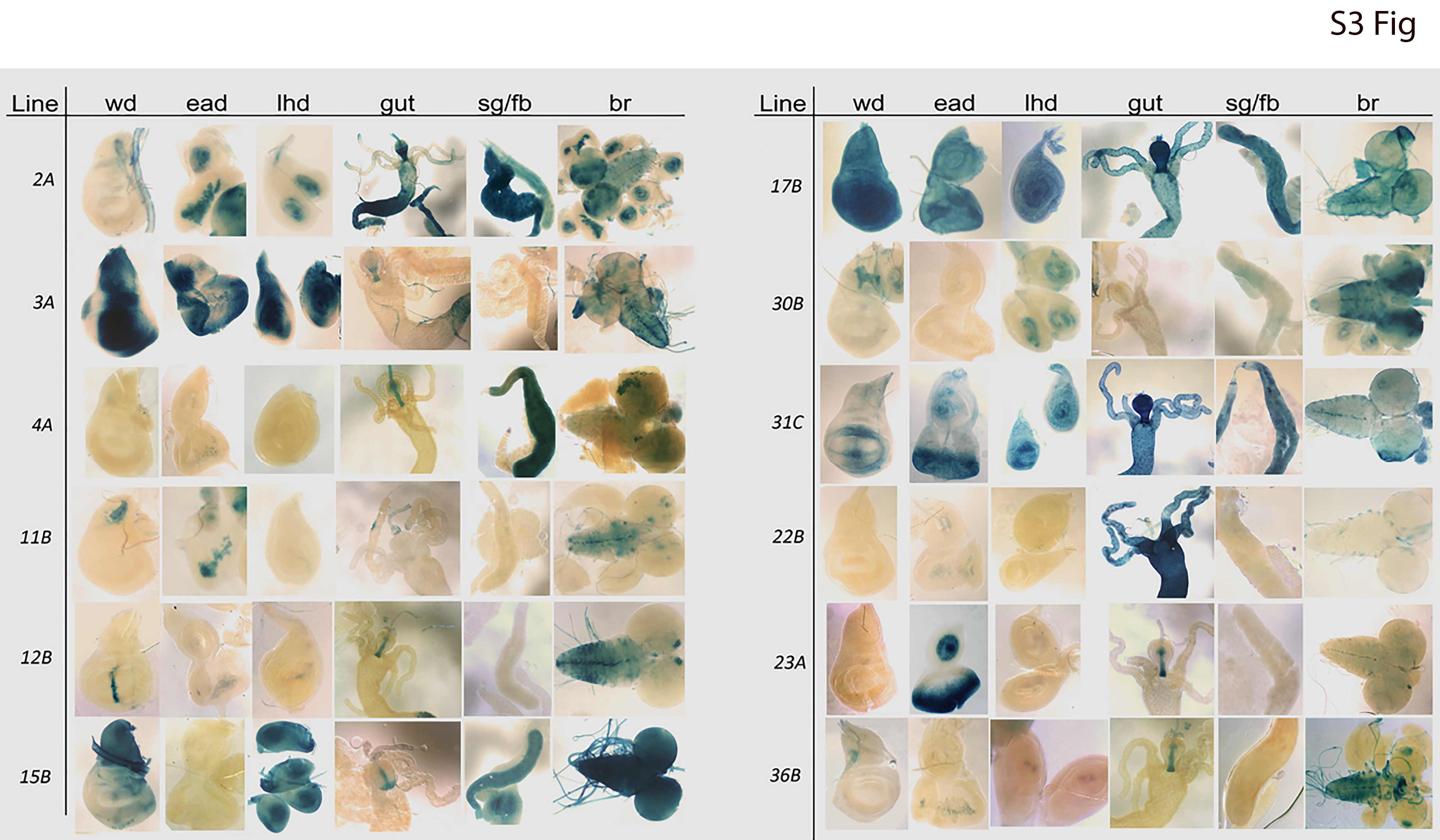


## Figure S3. Beta-gal activity induced in larval tissues by P*_tub_*-LexA-BAP170/SAYP in twelve out of twenty lines transgenic for the LexAop-LacZ reporter.

Wd, wing imaginal discs; ead, eye-antennal imaginal discs; lhd, leg-haltere discs; g, guts; sg/fb, salivary glands/fat bodies; br, larval brain.

##
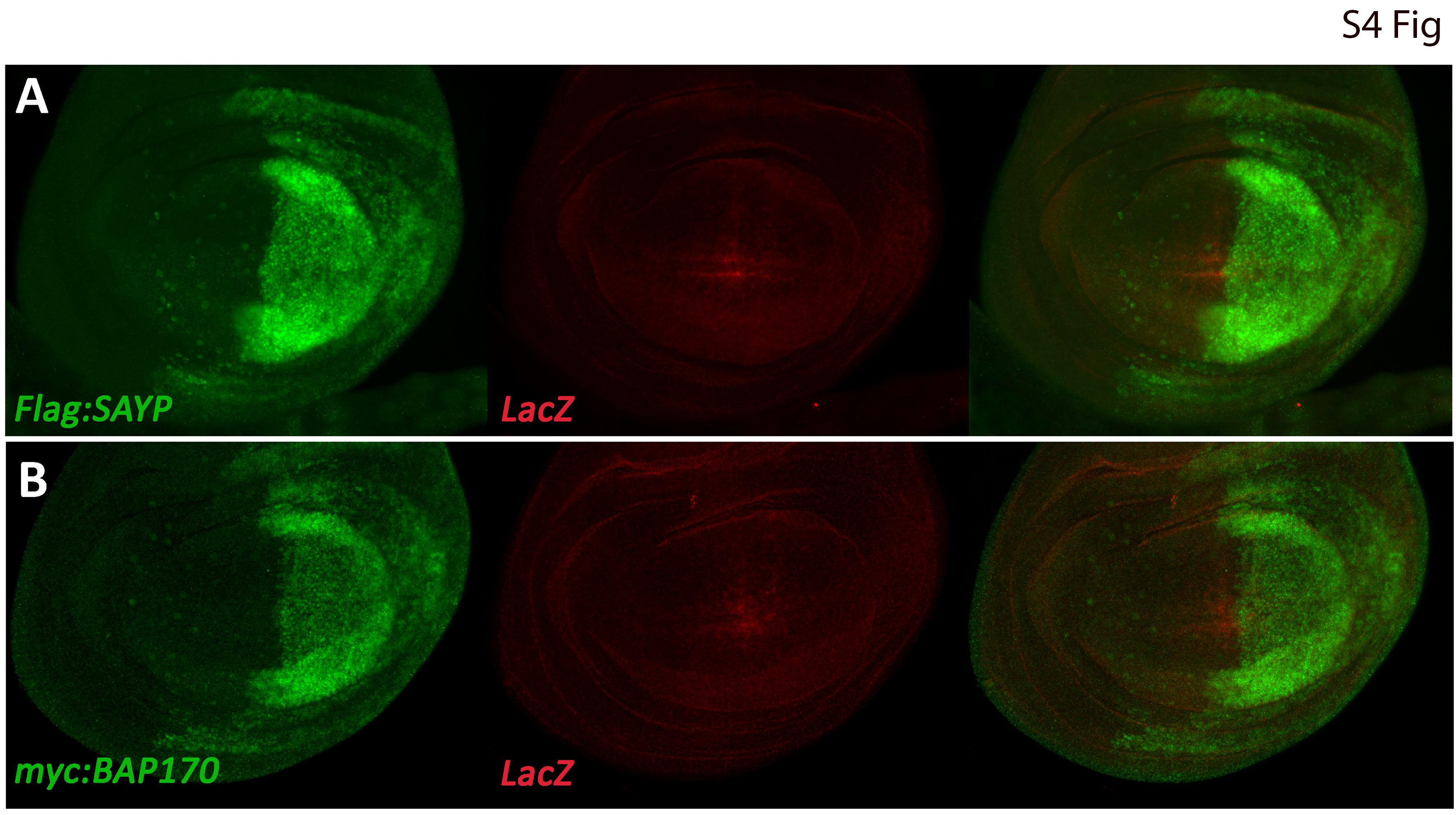


## Figure S4. Wild-type SAYP or BAP170 overexpression cannot induce enhancer-dependent LexAop-LacZ expression.

Immuno-fluorescence imaging of wild type SAYP (A) or BAP170 (B) tagged with FLAG or myc epitopes, respectively, expressed from the UAS transgenes under the en-GAL4 driver in the wing discs of *LexAop-LacZ^Dad^* transgenic larvae. Expression of the LacZ reporter is not activated by overexpression of SAYP or BAP170 in posterior cells, but remains at its very weak background level. The genotypes are: *UAS-3xFLAG:SAYP/en-GAL4; LexAop-LacZ^Dad^* (A), *UAS-myc:SAYP/en-GAL4; LexAop-LacZ^Dad^* (B).


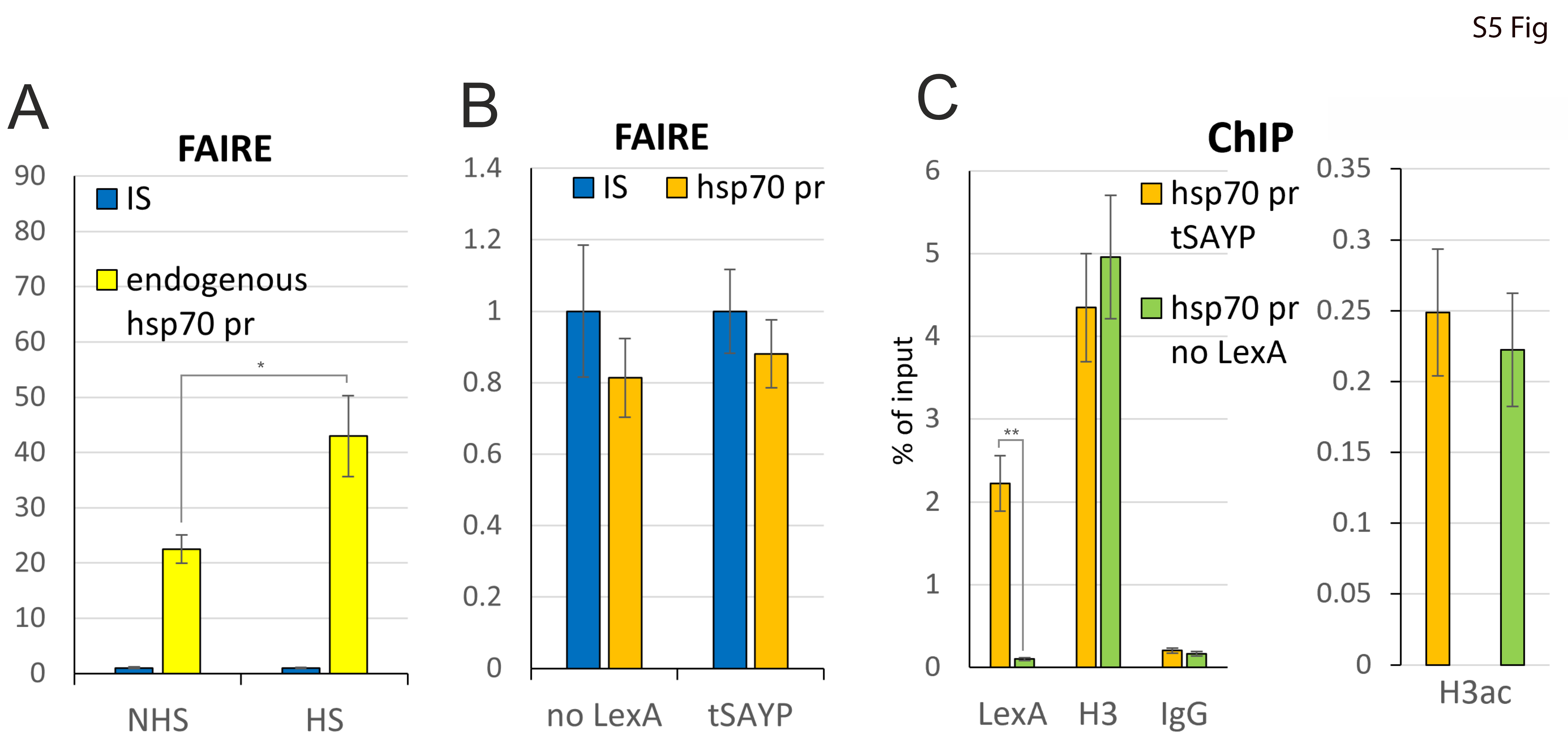


## Figure S5. Analysis of the chromatin state.

(A) FAIRE signal at the endogenous *hsp70* promoter relative to the control region (intergenic spacer, IS), which was set as 1. Larvae grown at a normal temperature (NHS) or after 30-minute treatment at 37° (HS) were tested. (B) FAIRE signal at the transgene *hsp70* promoter relative to the control region (intergenic spacer, IS), which was set as 1. Control flies (no LexA) and flies expressing LexA:SAYP fusion were tested. (C) ChIP analysis of the *hsp70* reporter promoter in the presence or absence of tSAYP. Antibodies used are indicated at the bottom (LexA fusion; H3, histone H3; H3ac, acetylated H3). The level of a factor is shown as a percentage of Input, H3ac was normalized to H3. *P < 0.05 and **P < 0.01.

##
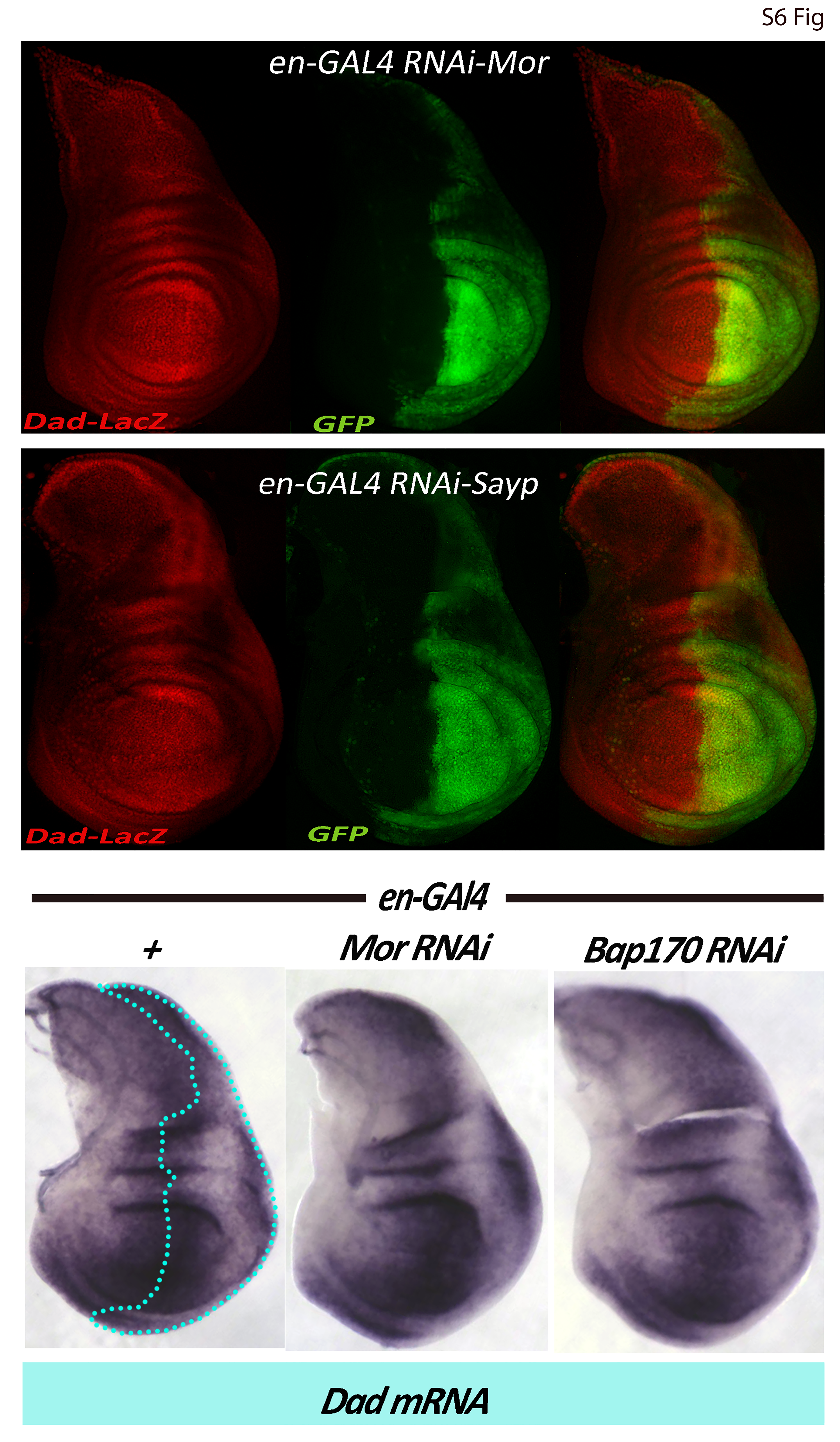


## Figure S6. *Dad* expression is independent of PBAP.

*Dad* enhancer activity is not affected by depletion of MOR (top) or SAYP (middle). *Dad* transcription (bottom panel) is independent of MOR or BAP170 as demonstrated by in situ hybridization with a *Dad* probe. *Dad* enhancer activity was detected using the enhancer trap line *P{lacW}Dad^j1E4^*. Genotypes: (top) *en-Gal4,UAS-GFP/+; P{lacW}Dad^j1E4^/UAS-RNAi-Mor[Vdrc6969]*; (middle) *en-Gal4,UAS-GFP/UAS-RNAi-SAYP [Vdrc105946], P{lacW}Dad^j1E4^*; (bottom) wild type (left); *en-Gal4/+; UAS-RNAi-Mor [Vdrc6969]* (center); *en-Gal4/+, UAS-RNAi-BAP170 [Vdrc34582]* (right).

##
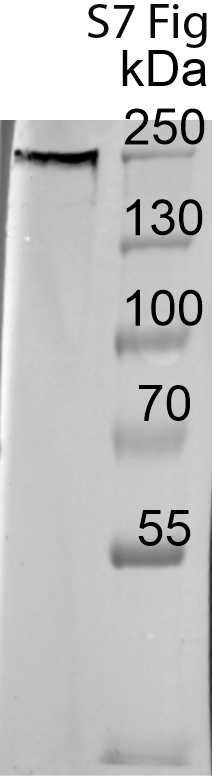


## Figure S7. Western blot analysis of a nuclear embryonic extract with a-BRM antibody.

## Supplementary Table 1

UAS-RNAi lines used in this work. Effects on LexA-SAYP/LexA-BAP170 mediated activation of the *LexAop-LacZ^Dad^* responder are indicated (↓, downregulated; ↑, upregulated; NE, no effect; ND, not determined). All lines except UAS-RNAi-Polybromo cause an evident phenotype upon activation with en-Gal4 (from larval/prepupal lethality to defects in adult wing morphology, columns 7 and 8). Phenotypes induced with other GAL4 lines are described in the last column, with references.

|  | *Affected*  *Gene* | *RNAi Line* | Effect on *LexA:Sayp, LexAop-LacZ^Dad^*  expression | Effect on *LexA:Bap170, LexAop-LacZ^Dad^*  expression | Effect on Dad expression | Phenotype with  engrailed-GAL4  (this work) | Phenotype with  tubulin-GAL4  (this work) | Phenotypes described with other Gal4 lines |
| --- | --- | --- | --- | --- | --- | --- | --- | --- |
| PBAP COMPLEX | *mor* | *VDRC6969* | ↓ | ↑  (Discussed in the text) | N.E. | Prepupal lethal | larval/prepupal lethal | pnr-GAL4^a^  Bx-MS1096-GAL4^b^  salm-GAL4^c^  *C564‐Gal4 and Hml‐Gal4^e^*  http://flybase.org/reports/FBal0210097.html |
|  | *bap170* | *VDRC34582* | ↓ | ↓ | N.E. | Adult wing extraveins | prepupal lethal | pnr-GAL4^a^  salm-GAL4^c^  http://flybase.org/reports/FBal0198822.html |
|  | *brm* | *VDRC37721* | ↓ | N.E. | N.E. | Adult wing defects | larval/prepupal lethal | pnr-GAL4^a^  salm-GAL4^c^  insc-GAL4^d^  http://flybase.org/reports/FBal0209177.html |
|  | *e(y)3*  *(SAYP)* | *VDRC105946* | ↓ | N.E. | N.E. | Adult wing defects | larval/prepupal lethal | - |
|  | *polybromo*  *(PB)* | *VDRC108618* | N.E. | N.E. | N.E. | wt | viable | C564‐Gal4 and Hml‐Gal4^e^  bab1-Gal4^f^  http://flybase.org/reports/FBal0231490.html |

1. Mummery-Widmer, J.L.; Yamazaki, M.; Stoeger, T.; Novatchkova, M.; Bhalerao, S.; Chen, D.; Dietzl, G.; Dickson, B.J.; Knoblich, J.A. Genome-wide analysis of Notch signalling in Drosophila by transgenic RNAi. Nature 2009, 458, 987-992, doi:10.1038/nature07936.
2. Dietzl, G.; Chen, D.; Schnorrer, F.; Su, K.C.; Barinova, Y.; Fellner, M.; Gasser, B.; Kinsey, K.; Oppel, S.; Scheiblauer, S., et al. A genome-wide transgenic RNAi library for conditional gene inactivation in Drosophila. Nature 2007, 448, 151-156, doi:10.1038/nature05954.
3. Terriente-Felix, A.; de Celis, J.F. Osa, a subunit of the BAP chromatin-remodelling complex, participates in the regulation of gene expression in response to EGFR signalling in the Drosophila wing. Dev Biol 2009, 329, 350-361, doi:10.1016/j.ydbio.2009.03.010.
4. Neumuller, R.A.; Richter, C.; Fischer, A.; Novatchkova, M.; Neumuller, K.G.; Knoblich, J.A. Genome-wide analysis of self-renewal in Drosophila neural stem cells by transgenic RNAi. Cell Stem Cell 2011, 8, 580-593, doi:10.1016/j.stem.2011.02.022.
5. Bonnay, F.; Nguyen, X.H.; Cohen-Berros, E.; Troxler, L.; Batsche, E.; Camonis, J.; Takeuchi, O.; Reichhart, J.M.; Matt, N. Akirin specifies NF-kappaB selectivity of Drosophila innate immune response via chromatin remodeling. EMBO J 2014, 33, 2349-2362, doi:10.15252/embj.201488456.
6. He, J.; Xuan, T.; Xin, T.; An, H.; Wang, J.; Zhao, G.; Li, M. Evidence for chromatin-remodeling complex PBAP-controlled maintenance of the Drosophila ovarian germline stem cells. PLoS One 2014, 9, e103473, doi:10.1371/journal.pone.0103473.

## Supplementary materials

## Primers for PCR amplification and cloning of the *Dad* and *dpp* enhancers

JFL-attB [DadEnh-lexAop-hsp70-LacZ], 5’-cgAAGCTTTCGAGAGCGCCTTCAATTCTAATTC and 5’-cgAAGCTTTTCACCAACGGACAGCCGACGC

JFL-attB [lexAop-hsp70-LacZ-DadEnh], 5’-cgGAATTCTCGAGAGCGCCTTCAATTCTAATTC and 5’- cgGAATTCTTCACCAACGGACAGCCGACGC

JFL-attB [dppEnh-lexAop-hsp70-LacZ], 5’- cgAAGCTTTTCCACTCACCTTGTCAGCCAG and 5’- cgAAGCTTATTCCAGTGCTGGGAACGTG

JFL-attB [lexAop-hsp70-LacZ-dppEnh], 5’- cgGAATTCTTCCACTCACCTTGTCAGCCAG and 5’- cgGAATTCATTCCAGTGCTGGGAACGTG

## Primers used in ChIP and FAIRE analysis

point 1 (*hsp70* reporter promoter), 5’-TAGCGCTAGCGACGTCGAG and 5’-GCTTAGCGACGTGTTCACTTTG

point 2, 5’-TGTGCGATATATGGCTTTTGAAGTG and 5’-TGTAATCCAGTGAACAATAGAGAGAGCTG

point 3 (*Dad* enhancer), 5’-AGGTGAGCGTGTGTTGGTGTG and 5’-TCATACATACAGAATGCTTGCGTGC

point 4, 5’-TTGCCCATACCGAAAAATCCTG and 5’-TCAGCTACAGTGAAGCGTCTCAAAC

rDNA, 5’-AATTCAGAACTGGCACGGACTTGG and 5’-AGAGCACTGGGCAGAAATCACATTG

*hsp70* endogenous promoter, 5’-ACATACTGCTCTCGTTGGTTCG and 5’-TTGAATTGAATTGTCGCTCCGTAG

intergenic spacer (IS), 5’-GCCCACAATCGGACATTGAC and 5’- TCCCACTCCCAAGTCAGGC

## Primers and probes used in 3C analysis

Anchor point (*hsp70* reporter promoter), 5’-CAAAGTGAACACGTCGCTAAGC

Point I (intergenic spacer), 5’-GGACACACACGCTACTCAGAT

Point II (*lacZ* reporter), 5’-ACGCCATCCCGCATCTG

Point III (*Dad* promoter), 5’-CGTCGTAAACAACAAACAAGTGC

Point IV (*Dad* gene), 5’-ATACGGTTTATGTGGAGGGAATGAA

Point V (*Dad* enhancer), 5’-TTCCCCCTTACCAATCGTTCC

Point VI (*Dad* gene), 5’-CCAGACGCAAACATAGACACC

TaqMan probe, 5’-(FAM)TAACCAGCAACCAAG(T-BHQ1)AAATCAACTGCAACT-P

*RpII* forward primer, 5’-GGGGCGACCAGAAGAAGGC

*RpII* reverse primer, 5’-GCGATGGCAGAAGGAGCAAT

TaqMan *RpII* probe, 5’-(FAM)TCAAGCGAT(T- BHQ1)CAACACCTGGGAGACACCG-P
