# Supplementary figures and images for: Subunits of the PBAP Chromatin Remodeler Are Capable of Mediating Enhancer-Driven Transcription in Drosophila
Source: Int J Mol Sci. 2021 Mar 11;22(6):2856. doi: 10.3390/ijms22062856 (PMC7999800; doi:10.3390/ijms22062856)

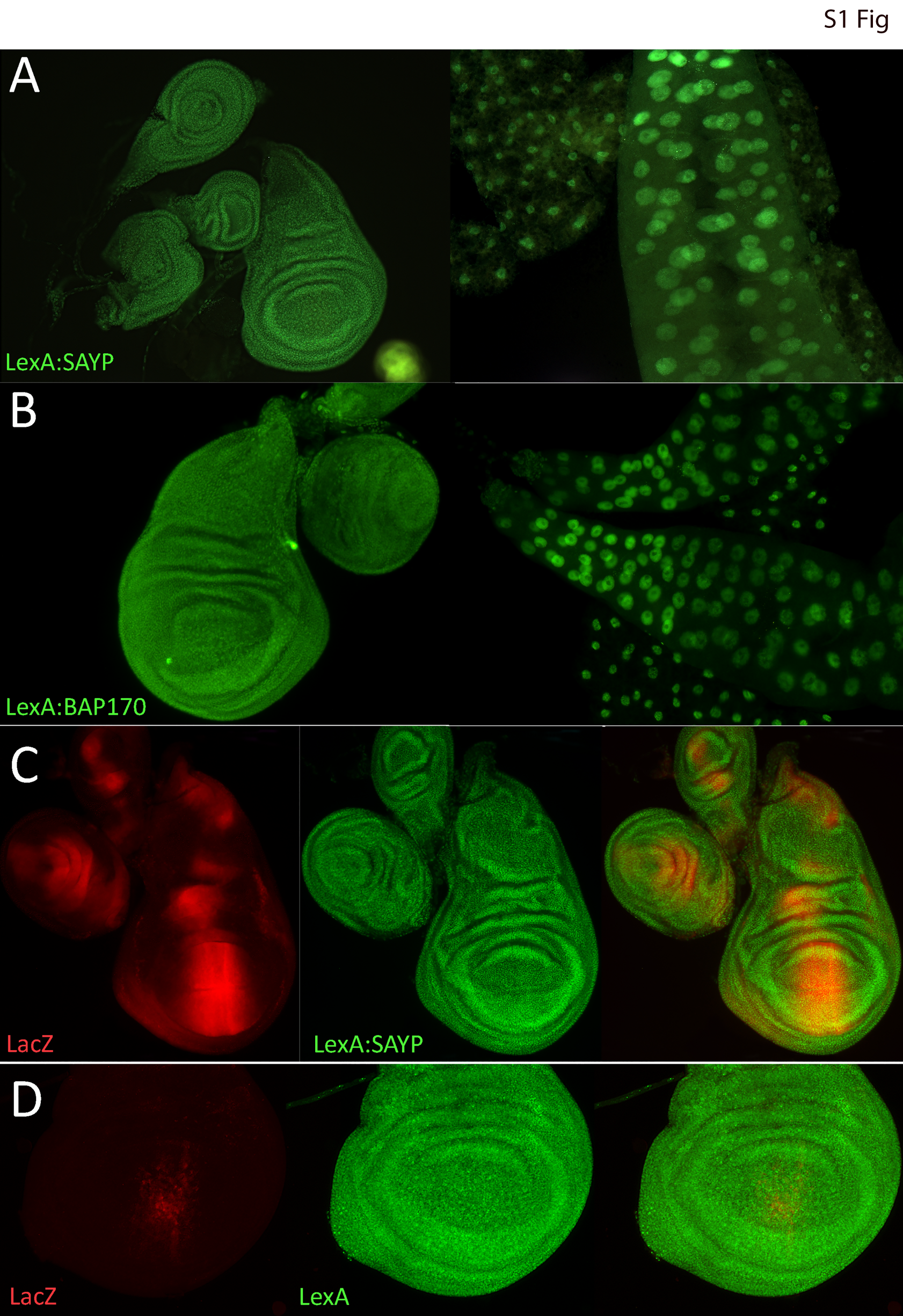

Supplement: Supplementary file 1 [file ijms-22-02856-s001.zip › S1_Fig.tif]

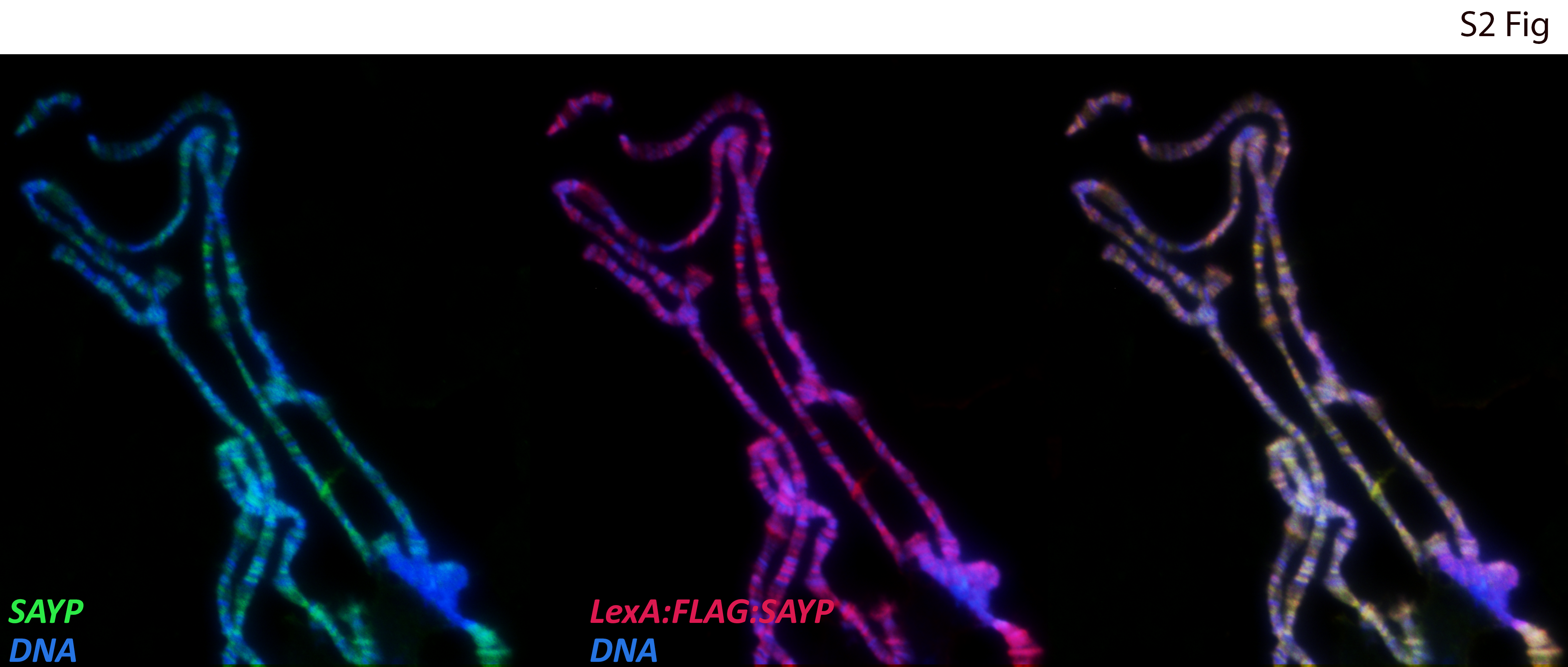

Supplement: Supplementary file 1 [file ijms-22-02856-s001.zip › S2_Fig.tif]

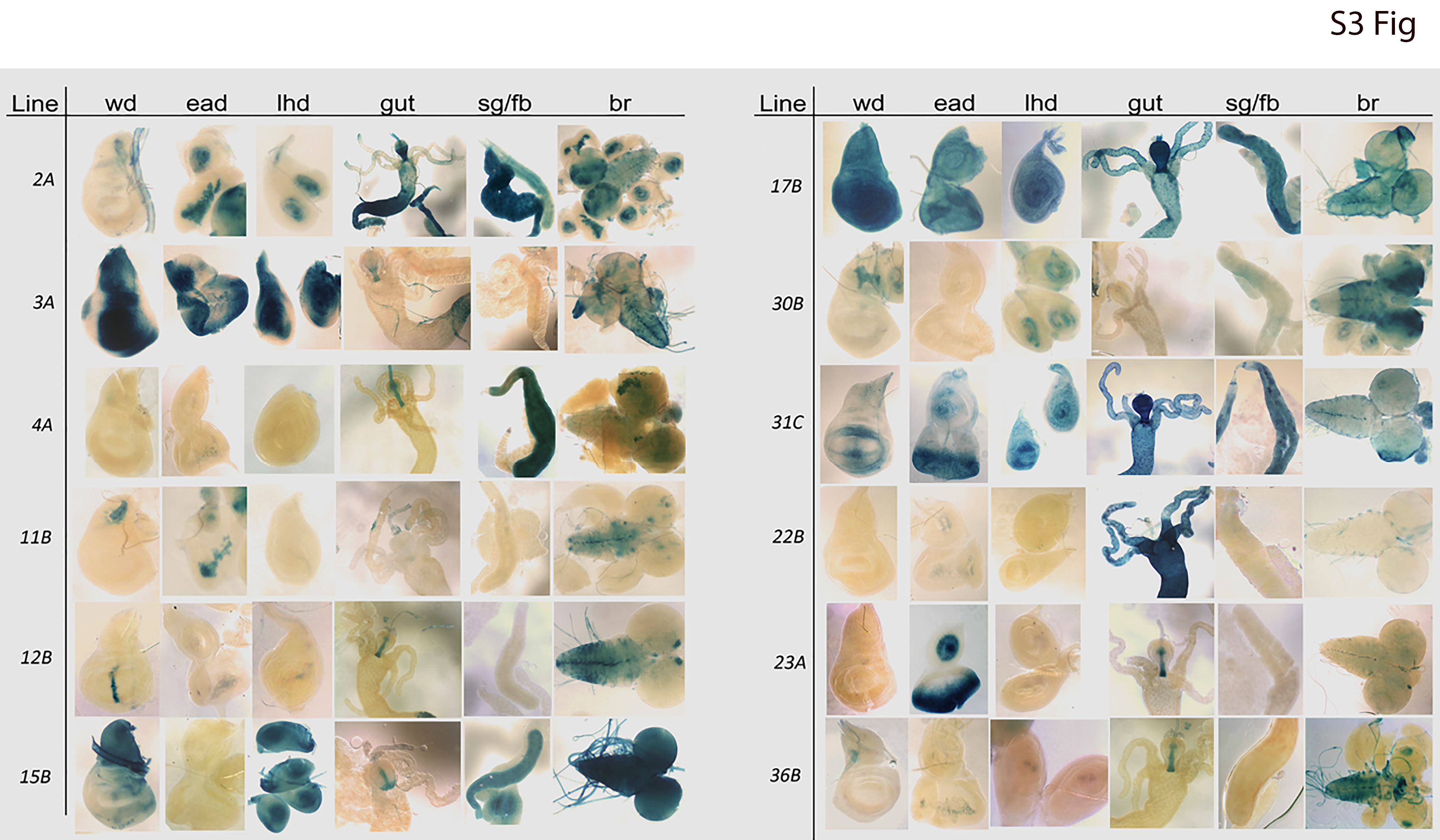

Supplement: Supplementary file 1 [file ijms-22-02856-s001.zip › S3_Fig.tif]

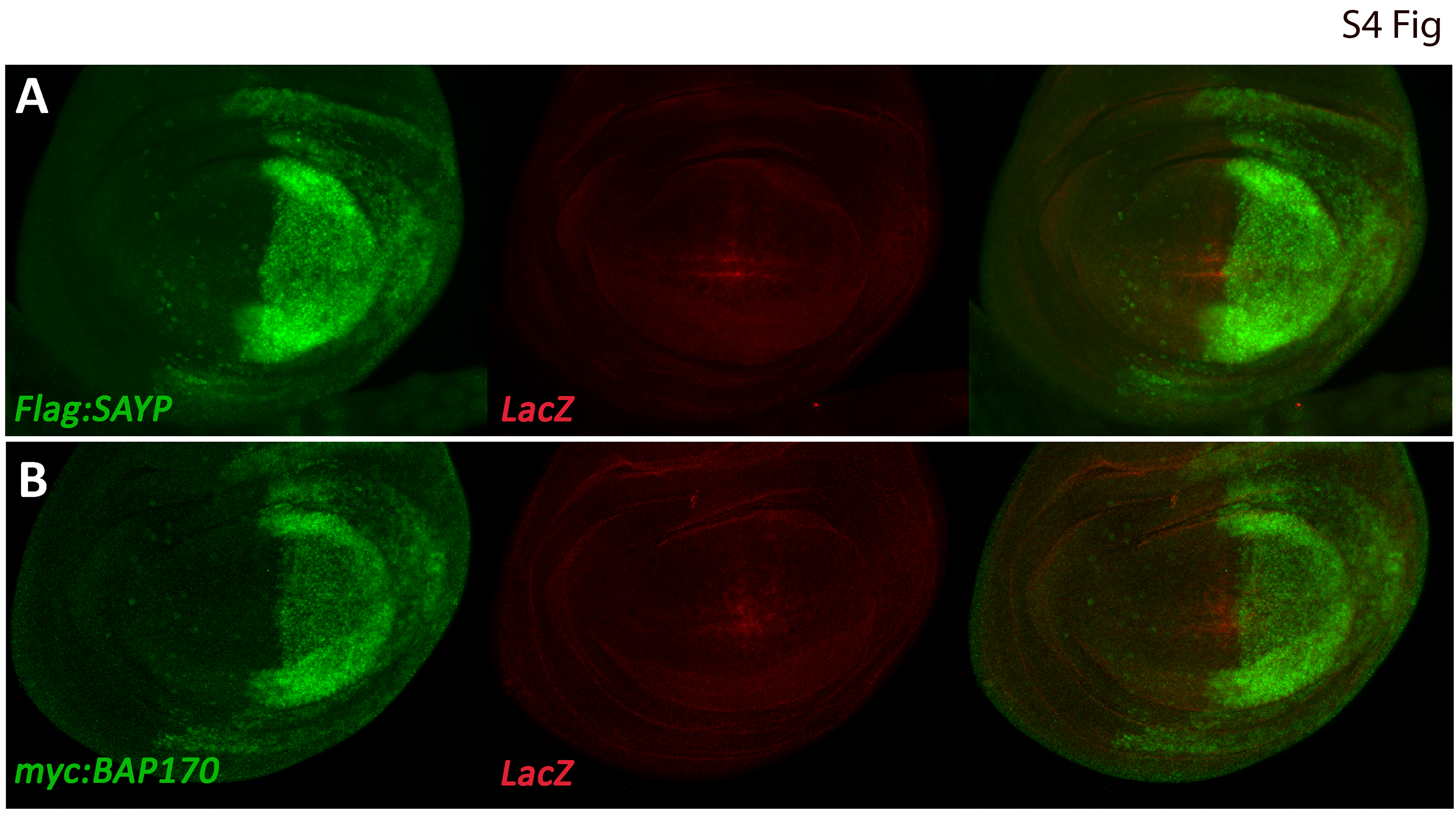

Supplement: Supplementary file 1 [file ijms-22-02856-s001.zip › S4_Fig.tif]

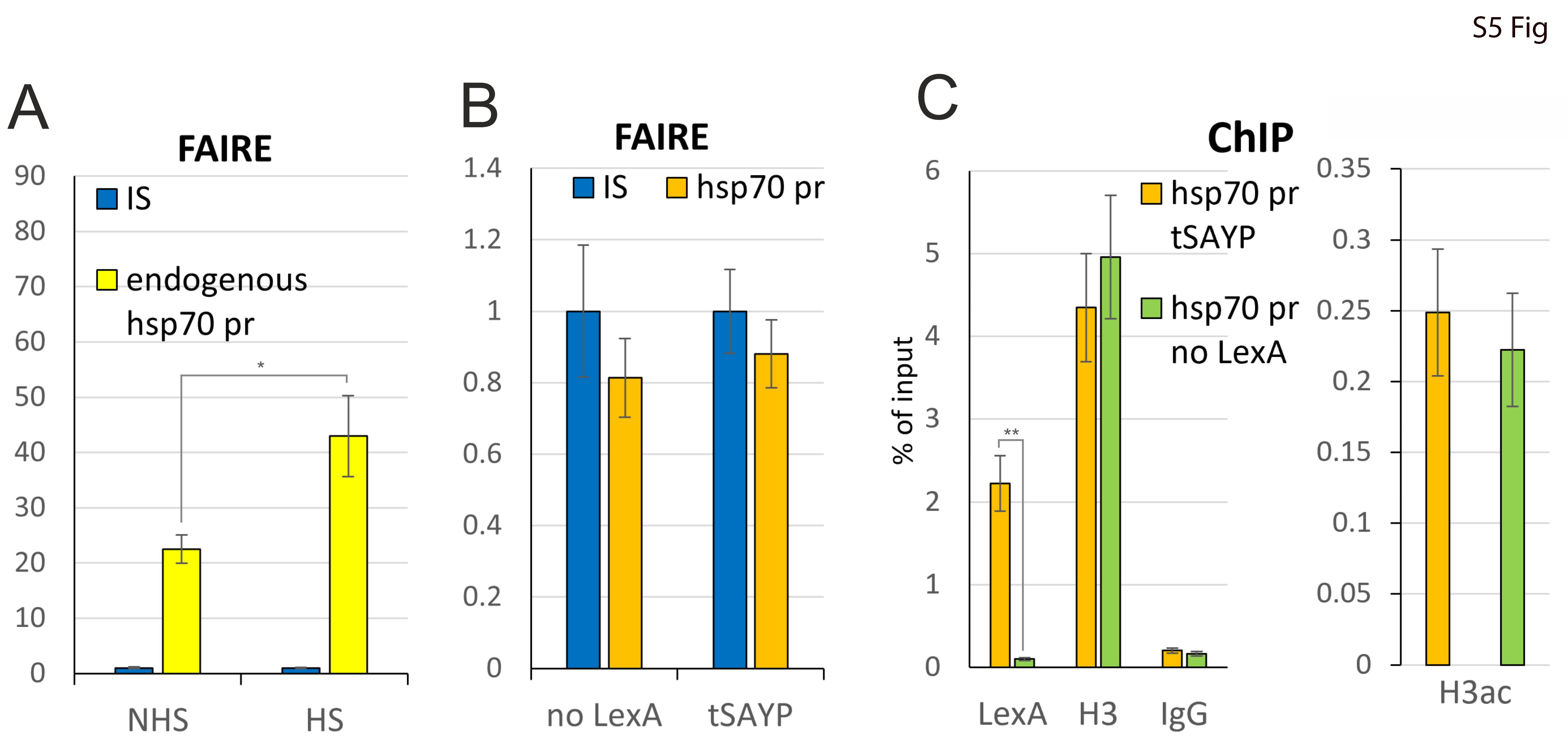

Supplement: Supplementary file 1 [file ijms-22-02856-s001.zip › S5 Fig.tif]

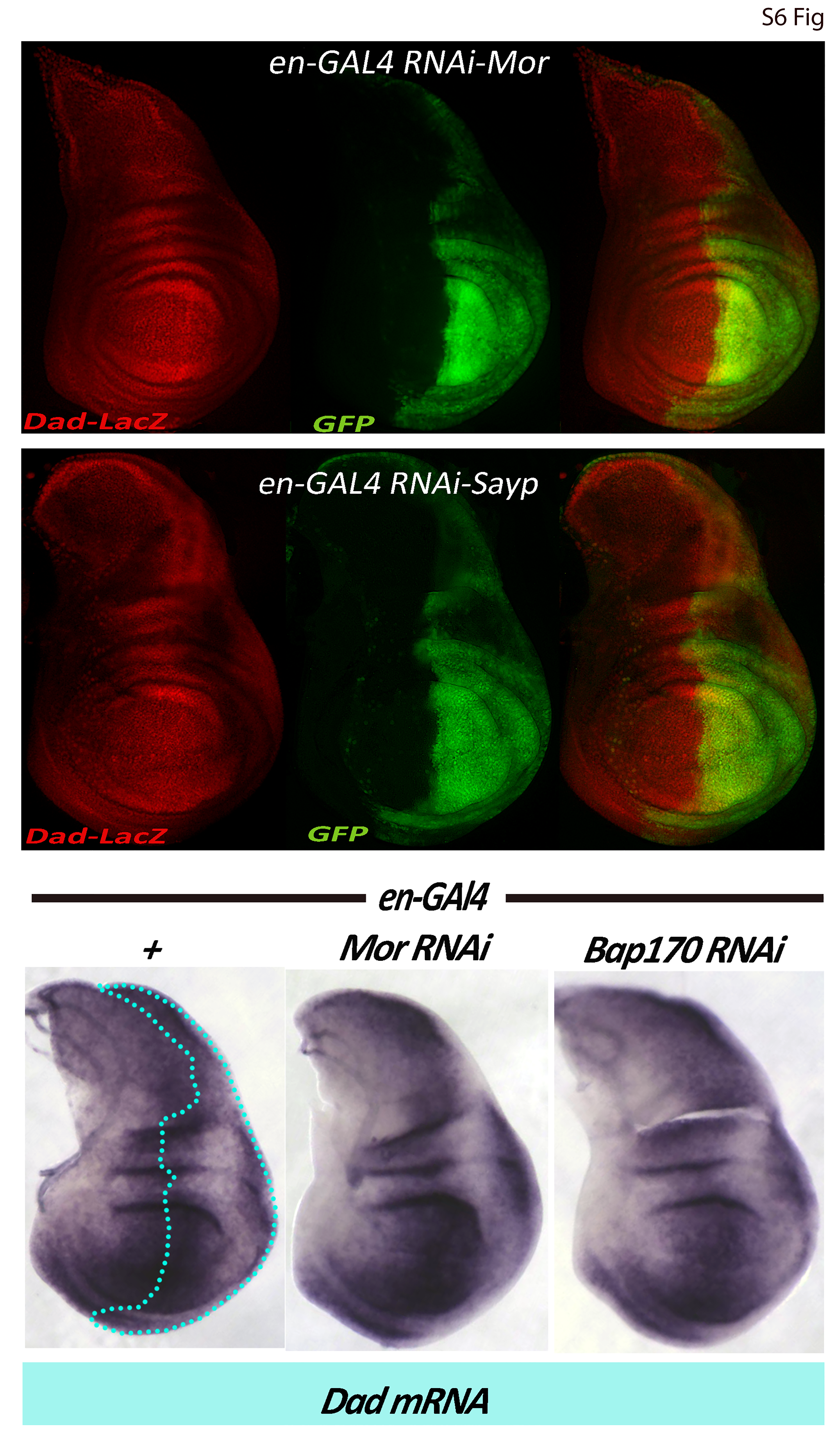

Supplement: Supplementary file 1 [file ijms-22-02856-s001.zip › S6_Fig.tif]
